# Gap Detection for Genome-Scale Constraint-Based Models

**DOI:** 10.1155/2012/323472

**Published:** 2012-09-10

**Authors:** J. Paul Brooks, William P. Burns, Stephen S. Fong, Chris M. Gowen, Seth B. Roberts

**Affiliations:** ^1^Center for the Study of Biological Complexity, Virginia Commonwealth University, P.O. Box 843083, Richmond, VA 23284, USA; ^2^Department of Statistical Sciences and Operations Research, Virginia Commonwealth University, P.O. Box 843083, Richmond, VA 23284, USA; ^3^Department of Chemical and Life Science Engineering, Virginia Commonwealth University, P.O. Box 843083, Richmond, VA 23284, USA

## Abstract

Constraint-based metabolic models are currently the most comprehensive system-wide models of cellular metabolism. Several challenges arise when building an *in silico* constraint-based model of an organism that need to be addressed before flux balance analysis (FBA) can be applied for simulations. An algorithm called FBA-Gap is presented here that aids the construction of a working model based on plausible modifications to a given list of reactions that are known to occur in the organism. When applied to a working model, the algorithm gives a hypothesis concerning a minimal medium for sustaining the cell in culture. The utility of the algorithm is demonstrated in creating a new model organism and is applied to four existing working models for generating hypotheses about culture media. In modifying a partial metabolic reconstruction so that biomass may be produced using FBA, the proposed method is more efficient than a previously proposed method in that fewer new reactions are added to complete the model. The proposed method is also more accurate than other approaches in that only biologically plausible reactions and exchange reactions are used.

## 1. Introduction

Flux balance analysis (FBA) is the use of a linear program (LP) to model the flow of metabolites through the network of reactions in a cell [1]. FBA simulations give insight into the relative rates at which reactions occur when the cell is optimized for a specific objective. A fundamental assumption of FBA is that organisms can function optimally (often as a result of adaptive evolution) in that they make optimal use of scarce resources to serve the needs of the organism. This characterization of cell behavior naturally leads to a math programming modeling paradigm. FBA has been used to predict growth rates, gene essentiality, and other features of multiple organisms [2–5].

Several related challenges are encountered in the building of metabolic reconstructions. To apply FBA to a constraint-based model, both a reaction network (representing organism-specific biochemical capabilities) and an objective (representing a desired or measurable physiological goal) need to be specified. Currently, complete reaction networks for organisms are not known. There may be reactions in a cell that must be active for the production of biomass that have not been cataloged in biological databases or documented in the literature. Another challenge is modeler error; the modeler can mistakenly omit a reaction or transport process that is necessary for the production of biomass. Aside from establishing a model that can produce biomass, a common difficulty in using FBA models is that of finding a culture medium that can allow the *in silico *cell to send flux through the biomass reaction.

Several methods for restoring functionality in broken FBA models, those incapable of a desired level of flux through the biomass reaction, have been previously proposed. GapFind [6] is a procedure that determines which metabolites in a network cannot be produced, and GapFill [6] determines a minimal set of reactions to add from a universal database so that a specified set of metabolites may be produced. These optimization-based procedures have already been integrated into the Model SEED metabolic reconstruction pipeline with some success [7]. Reed et al. [8] utilize a method that adds a minimum-sized set of reactions from a universal database that allows for a specified level of biomass production in the resulting model. MetaFlux [9] is an automated approach to find missing reactions, exchange reactions, and biomass metabolites. OptStrain [10] determines the maximum possible yield of a desired product based on the inclusion of all reactions in a universal database and then finds the minimum number of reactions from the database needed to achieve the optimal yield. Segrè et al. [11] use the Forward Propagation and Backward Propagation/Backtracking algorithms [12] to first determine the metabolites that can be produced in a model, and then find the precursors of essential nonproducible metabolites that cannot be produced.

Several investigators have proposed methods for filling gaps in metabolic networks outside of the FBA paradigm, including searching through a network of metabolites and reactions for logically possible paths [13, 14] and using logic programming to construct pathways [15]. These methods do not ensure that the mass balancing constraints of FBA models are satisfied, nor do they consider the effects of generated pathways on the production of biomass. Thus, the application of these methods does not guarantee the generation of a constraint-based model that produces biomass when FBA is applied.

A fundamental assumption of FBA modeling is that metabolites remain at constant concentration within the cell. Throughout this paper, we use the term *metabolite *to refer to any molecule whose concentration is of interest, including byproducts of metabolism, coenzymes, and protons. Let *v*_*j*_ be the flux through reaction *j*, for each *j* ∈ *R*, which is the number of times that a reaction occurs per unit time. Let *S*_*ij*_ be the stoichiometric coefficient for metabolite *i* in reaction *j*, for each *i* ∈ *M* and *j* ∈ *R*, with the convention that *S*_*ij*_ is negative for molecules *i* that are reactants for reaction *j*, positive for metabolites *i* that are products for reaction *j*, and 0 otherwise. Metabolites may participate in a unidirectional or reversible *exchange reaction*. For our purposes, it will be helpful to distinguish *source reactions* from *escape reactions* and assign variables *b*_*i*_^src^ and *b*_*i*_^esc^ for the fluxes through these reactions. We wish to restrict transport fluxes to zero for any metabolite unless its concentration changes in the cell due to transport processes. The conservation of mass for metabolite *i* may be stated as follows:

(1) $$\begin{array}{c} \sum_{j\in R}S_{ij}v_{j}+b_{i}^{\text{src}}-b_{i}^{\text{esc}}=0. \end{array}$$

The set of reactions *R* may include a (potentially artificial) *biomass* reaction which reflects the objective of the cell in terms of which metabolites are emphasized for production or consumption by other processes. The objective

(2) $$\begin{array}{c} \max v_{\text{biomass}} \end{array}$$

can be added to the model, reflecting the desire to maximize flux through the biomass reaction. Maximizing flux through the biomass reaction is one of several possible objectives that one could assign to a cell. FBA models with this particular objective have been shown to reflect the behavior of single-celled organisms during cell growth. Assessing whether positive biomass production is possible is an effective method for testing the completeness of a metabolic reconstruction. If an FBA model is incapable of producing biomass, then there is likely a gap in the reaction network.

Upper and lower bounds on each reaction flux are specified. If possible, these bounds are based on experimentally observed fluxes and free energy considerations, as for the *S. cerevisiae *and *E. coli *models [16–18]. If not, then a common lower and upper bound for all reactions can be assigned, and the fluxes returned by FBA give the investigator an idea of the relative activity of the reactions in the network for a given biomass reaction; the actual flux values in this latter case are less important than the ratios. For example if *v*_*j*_/*v*_*k*_ ≥ 4, the model indicates that a mechanism for maximizing biomass production exists wherein reaction *j* is at least 4 times as active as reaction *k*. If we generate (1) for metabolites and reactions within a cell and add the flux bounds, we obtain the linear programming-based FBA model. The general model can be expressed compactly as follows:

(3) max⁡vbiomassSv+bsrc−besc=0,L≤v≤U,Lsrc≤bsrc≤Usrc,Lesc≤besc≤Uesc.

In this paper, we propose a new approach to address the challenges of building FBA models called FBA-Gap. The procedure identifies gaps in the metabolic network that are preventing flux through a specified objective, which in our case is the biomass reaction that represents cellular growth. Given a metabolic reconstruction and a biomass reaction, the goal is to find the most plausible modification of the metabolic reconstruction so that the model is capable of sending flux through the biomass reaction. FBA-Gap uses mathematical optimization to determine a minimum cost set of additional exchange reactions needed such that the flux through the biomass reaction can exceed a given threshold. Costs are assigned to source and escape reactions *a priori *based on their plausibility and distance to the biomass reaction. In general, exchange reactions for metabolites that exist in the extracellular compartment are given a low cost, while exchange reactions for metabolites that exist only in cytosolic and intracellular compartments are given a high cost. The output is a minimum cost set of exchange reactions and a flux distribution for the expanded reaction network. If the model is robust and has no detrimental gaps, the selected exchange reactions will correspond to missing transport reactions for uptake of metabolites from *in silico* culture medium or for discharge of byproducts into the extracellular space. However, if the model has internal gaps in the reaction network, exchange reactions will be added for internal metabolites that are furthest from the biomass reaction. 

 Our method is a departure from previous gap-filling methods in that we place an increased emphasis on the accuracy of the final model. The approach is to preserve the set of reactions in the initial model and to direct the model builder to a set of reactions that lead to a biomass-producing model and can be added with high confidence. In the GapFind/GapFill framework, reactions are added until *every *metabolite in the model is produced, and many additional reactions may be added to a model that are not required for the production of biomass. We will demonstrate that the proposed method is less computationally intensive than GapFind/GapFill. In the method described in [8], hereafter referred to as GapReed, reactions may be added to the model which are downstream/upstream of the actual gap. In other words, there is no attempt to ensure that modifications address gaps in the “backbone” of the network; the gaps may be masked by implausible exchange reactions or secondary pathways. The emphasis in our method is directing the modeler to the gaps in the backbone of the network that can be addressed by adding high-confidence reactions to the model.

The cost structure in FBA-Gap for the artificial exchange reactions is crucial to the proper identification of gaps in the metabolic network. Our approach is to identify the gaps that are furthest distance from the biomass reaction, utilizing as much of the existing network as possible. A trivial “fix” to any constraint-based model would be to add exchange reactions for every component of the biomass reaction, which would always result in a solution that has no biological relevance. Measuring distance in a metabolic network is a well-studied problem. Distances between metabolites in a metabolic network have been used to establish and refute scale-freeness [19, 20]. Investigators have noted difficulties associated with the inclusion of coenzymes in distance calculations, not the least of which is specifying which metabolites are coenzymes [13]. Some of these coenzymes are ubiquitous so that every metabolite appears near every other metabolite. Solutions to these difficulties include the introduction of compartments [13], excluding the most common metabolites from distance calculations [21], and using the Euclidean distance of attribute vectors for metabolites [14]. In FBA-Gap, the length of a path in the metabolic network is based on the number of reactions in which each metabolite occurs, penalizing paths that pass through often-occurring metabolites. Gaps where coenzymes play a prominent role can be discovered, but preference is given to other gaps. 

In the remainder of the paper, we describe the FBA-Gap method for building metabolic reaction networks and demonstrate its effectiveness in computational experiments. The method is used to help create a new metabolic reconstruction for a cellular organism based on a partial reconstruction. We compare the accuracy and computation time of FBA-Gap to existing gap-filling methods for this model. We then remove the exchange reactions from several existing models of organisms and apply FBA-Gap, yielding a hypothesis for minimal media for each organism. Finally, we delete a portion of the internal reactions of a working model, and apply FBA-Gap to detect the resulting gaps in the network.

## 2. Materials and Methods

**Figure 1:**
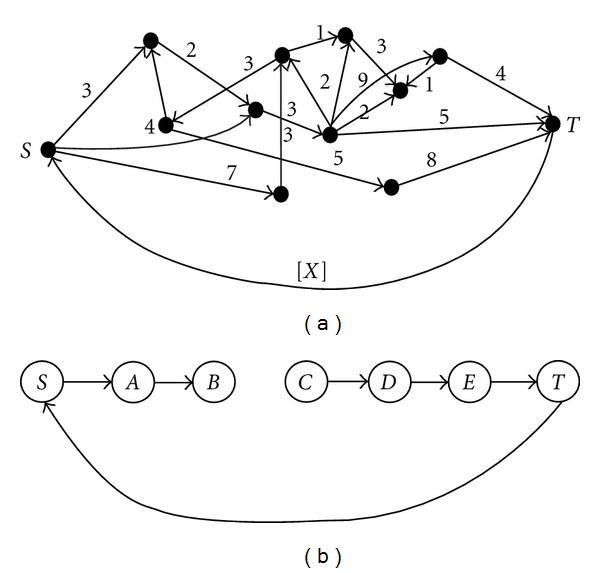
(a) An illustration of a maximum flow problem on a graph. The numbers above the arcs are capacities, and we wish to maximize flow from the source *S* to the sink *T*; equivalently, we wish to maximize flow along the artificial arc (*T*, *S*) such that the flow at each node is balanced. (b) An example of a small maximum flow problem with a gap such that no flow along arc (*T*, *S*) is possible.

FBA-Gap takes as input an FBA model and a lower bound for the flux through the artificial biomass reaction (to ensure growth). Whereas FBA can be considered a generalized maximum flow on a hypergraph, consider an analogy with maximum flows on graphs (Figure 1(a)). Intuitively, a gap corresponds to a missing arc. The main idea behind FBA-Gap is to find a minimum-cost set of artificial exchange reactions so that biomass may be produced. Note that for the graph in Figure 1(b), artificially adding flow to any of nodes *C*, *D*, or *E* will ensure positive flow along the artificial arc. Given that we would like to fill the gap, we would benefit the most by knowing the needed exchange reaction that is furthest from the biomass reaction. This desire leads us to define a notion of *distance from the biomass reaction* and a corresponding cost structure that will lead us to the gaps.

**Integer Programming Model**

Let

(4) $$\begin{array}{c} x_{i}=\{\begin{array}{cc} 1 & \text{if}\;\text{a}\;\text{source}\;\text{reaction}\;\text{is}\;\text{added}\;\text{for}\;\text{metabolite}\;i\\ 0 & \text{o}.\text{w}. \end{array}\\ y_{i}=\{\begin{array}{cc} 1 & \text{if}\;\text{an}\;\text{escape}\;\text{reaction}\;\text{is}\;\text{added}\;\text{for}\;\text{metabolite}\;i\\ 0 & \text{o}.\text{w}. \end{array} \end{array}$$

for *i* ∈ *R*. Then a minimum-cost set of exchange reactions for which a minimum threshold of flux through the biomass is attained can be determined by solving the following mixed-integer program:

(5) min⁡ (csrc)Tx+(cesc)Ty,s.t.    Sv+bsrc−besc=0,   L≤v≤U,   (Lsrc)Tx≤bsrc≤(Usrc)Tx,   (Lesc)Ty≤besc≤(Uesc)Ty.

Note that a positive lower bound for the biomass reaction, *L*_biomass_, is specified in the set of flux lower bounds. The first constraint ensures that a valid flux distribution is derived, that is, the mass balance constraints are satisfied. The last two constraints ensure that if the flux along a exchange reaction is positive, then an appropriate cost is enforced. The remaining constraint contains bounds for the reactions fluxes. Solving (5) is shown to be *NP-Complete* (see in the Supplementary Material available online at doi:10.1155/2012/323472). The selection of exchange metabolites that are most biologically plausible and/or furthest from the biomass reaction is ensured by a cost structure that is described in the next section.

**Cost Structure for Exchange Reactions**

**Figure 2:**
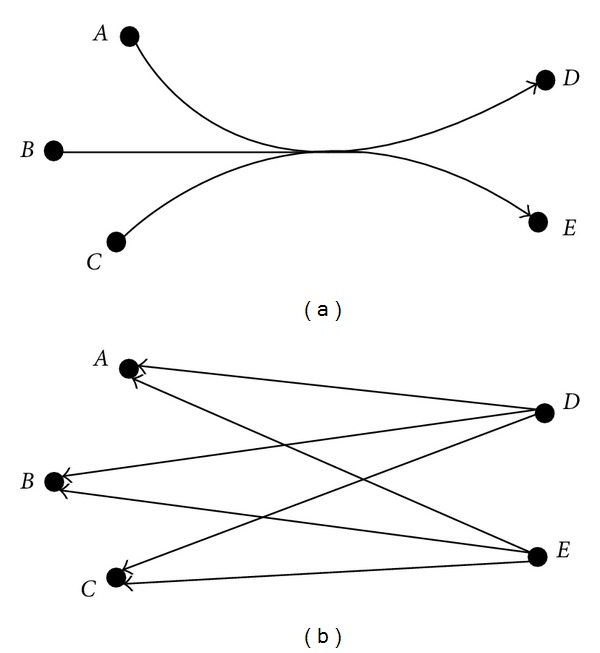
(a) An example of a hyperarc in the hypergraph H corresponding to a reaction *A* + *B* + *C* → *D* + *E*, and (b) the corresponding arcs in *G*, the graph used for calculating distances.

First, we assign costs to extracellular metabolites. Extracellular metabolites are substances that are either postulated to exist in the culture medium or are secreted by the cell. Adding a source reaction for such a metabolite is plausible if experimental culture media that support growth are likely to contain the substance, and adding an escape reaction is plausible if the cell likely secretes the metabolite. A low cost of 1 is assigned for biologically plausible exchange reactions for extracellular metabolites, and a cost of 20 is assigned for implausible exchange reactions for extracellular metabolites (Table S1). Therefore, up to 20 plausible exchange reactions will be selected before 1 implausible exchange reaction. The costs for artificial exchange reactions for internal metabolites are assigned based on the distance of a metabolite to the biomass reaction. Distance to the biomass reaction is defined as follows. Assume for the moment that all stoichiometric coefficients are 1. Let *H* = (*M*, *R*) where *R*⊆2^|*M*|^ × 2^|*M*|^ is the directed hypergraph associated with the reaction network for an organism, where each reaction corresponds to a hyperarc (Figure 2(a)). Define a directed graph *G* = (*M*, *ℛ*) as follows. For every hyperarc *r* ∈ *R* with tail nodes *T*_*r*_ and head nodes *H*_*r*_, and for every *i* ∈ *T*_*r*_ and *j* ∈ *H*_*r*_, there is an arc (*j*, *i*)∈*ℛ* (Figure 2(b)). An intuitive definition for the distance of a metabolite to the biomass reactants (products) is the minimum length of a directed path in *G* from the metabolite to a biomass reactant (product). This distance measure does not work well because, for example, a large proportion of reactions in a cell involve cofactors such as ATP. Every metabolite is either involved in a reaction where ATP is produced or consumed or will be near such a reaction by this distance measure. Therefore, every metabolite will appear to be near the biomass reaction.

To remedy this effect, we penalize paths that pass through these often-occurring cofactor metabolites. Instead of measuring graph distance by the number of arcs, we define the distance along an arc (*i*, *j*) in *ℛ* by *d* (*i*, *j*) = deg⁡ (*i*), where deg⁡ (*i*) is the degree of *i*. Note that the degree of node *i* in *G* is precisely the number of reactions in which metabolite *i* participates. The distance of a metabolite to the biomass reactants *d*_*i*_^src^ is the length of the shortest directed path in *G* to a biomass reactant, which can be determined by applying Dijkstra's algorithm [22]. The analogous distance to the biomass products is denoted *d*_*i*_^esc^. Let *d*_max⁡_^src^ (*d*_max⁡_^esc^) be the maximum distance among all metabolites with a directed path to the biomass reactants (products) in *G*. To penalize the source transport reactions that are near the biomass reactants, we define the cost for internal metabolite *i* to be *d*_max⁡_^src^ − *d*_*i*_^src^ + 20. The penalty of 20 in the cost formula ensures that the cost of an artificial exchange reaction for an internal metabolite is at least as high as the cost for an exchange reaction for an extracellular metabolite. Escape reactions are penalized in an analogous fashion. Dijkstra's shortest path algorithm is polynomial time and is computationally easy for the networks considered here. The computational complexity of the proposed method is dominated by solving instances of (5). We note here that our cost structure is less likely to find gaps involving ubiquitous but important backbone metabolites. However, the ubiquity of these metabolites in reactions that are already in the draft model indicates that they are unlikely to be responsible for a lack of biomass production in the *in silico* organism. We choose to penalize the inclusion of cofactors rather than simply removing them from the directed graph because determining which metabolites are cofactors can present a challenge [13]. The focus of the proposed method is on creating a high-confidence model that produces biomass, even if “secondary” pathways are involved; subsequent analyses with a working FBA model can help to identify remaining gaps in primary pathways.

**Applying FBA-Gap to Broken Models**

The trivial solution of zero flux on all reactions, including the biomass reaction, is always feasible for (3). A broken model is one for which the optimal objective value for (3) is lower than desired. The process of applying FBA-Gap to a broken model involves three stages: calculating distances to the biomass reaction, reviewing the output of FBA-Gap, and systematically adding reactions from a universal database. In the first stage, Dijkstra's algorithm is applied as described in the previous section to determine the distances of metabolites to biomass products and reactants. The distances are initialized to be infinite. If after application of the shortest path algorithm, the distance of a metabolite to biomass reactants, is still infinite, then there is no path in *G* to the biomass reactants for that metabolite, and the metabolite will never be selected by solving (5) as having a source reaction. By construction of *G, *there is no sequence of reactions in *H* beginning with a reaction that produces *i* and ending with a reaction that produces a biomass reactant. Therefore, adding a source reaction for *i* will only increase the objective value of (5) without helping to increase biomass production. Similarly, a metabolite *i* with *d*_*i*_^esc^ = *∞* after application of the shortest path algorithm will never be selected by solving (5) as having an escape reaction. The corresponding binary variables in (5) for these metabolites can be fixed to zero to reduce computation time. Further, the knowledgeable modeler can review this list to find metabolites that are known to be involved in the production of biomass and fill in gaps along known pathways.

The next stage includes the solution of the integer program (5). The problem is NP-hard and is related to the closed hemisphere problem (see Supplementary Material), suggesting that heuristics for the latter may be adapted to solve challenging instances. Action can be taken to reduce the computational time of solving the integer program directly. The modeler can fix the binary variables to 0 or 1 corresponding to exchange reactions that should not be eligible for selection and exchange reactions that should be selected, respectively. This feature can be used to allow the modeler to specify a particular carbon source for the cell or facilitate discovery of solutions corresponding to secretion of a particular substance. Specifying certain exchange reactions is analogous to determining the list of exchanges to be “tried” as in the MetaFlux procedure [9]. In FBA-Gap, if too many binary variables are fixed to zero, there is a risk that the integer program becomes infeasible. If a feasible solution exists, the output includes a set of exchange reactions, fluxes on those exchange reactions, and fluxes for all other reactions in the network that will provide the desired flux through the biomass reaction. If no feasible solution exists, then the minimum biomass flux must be reduced and/or the bounds on reaction fluxes in the network need to be expanded.

**Figure 3:**
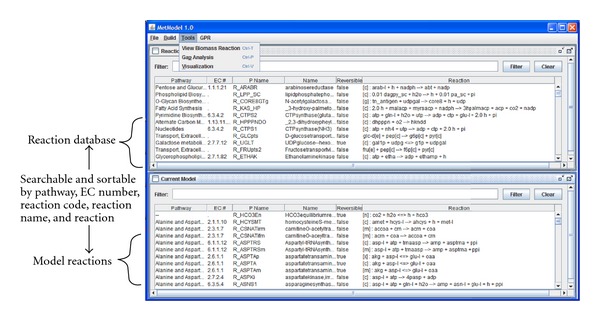
Screenshot of MetModel GUI, software for building FBA models. The top frame contains the universal reaction database, and the bottom frame contains the set of reactions in the current working model.

**Figure 4:**
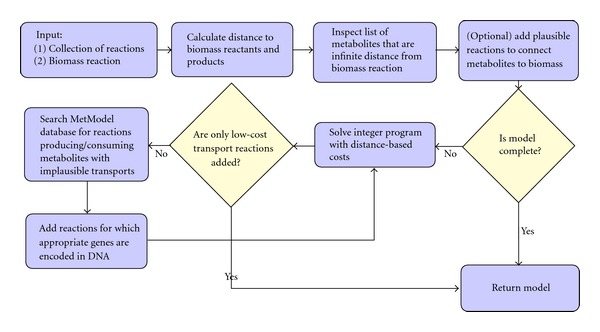
Flowchart of steps in building metabolic reconstructions using FBA-Gap. Determining if a model is complete involves checking if biomass is produced using only biologically plausible exchange reactions.

If a feasible solution contains only biologically plausible exchange reactions for extracellular metabolites, then the source reactions can indicate components of a culture medium for the organism. Biologically plausible exchange reactions are those that are for metabolites likely to exist in the culture medium and transportable across the cell membrane. If a feasible solution includes exchange reactions for internal metabolites (e.g., metabolites in the cytoplasm), then the selected exchange reactions give an indication of the location of gaps in the reaction network. The modeler can then consult the appropriate diagram in a publicly available biochemical pathway database, for example, KEGG [23], BioCyc [24], or Reactome [25]. The search for missing reactions is facilitated by the authors' software MetModel GUI (Figure 3). The software includes a searchable and sortable database of metabolic reactions that can be added to a model, as well as capabilities for searching the reactions in a user's model. After adding new reactions to the model, the integer program (5) is resolved and the process of adding reactions is repeated until the model uses only low-cost exchange reactions. A flowchart of the steps in FBA-GAP is depicted in Figure 4.

**Small Example**

**Figure 5:**
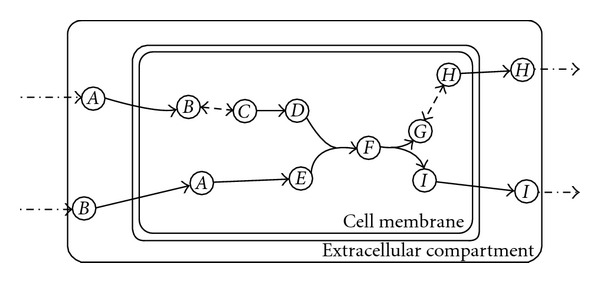
An example of a “broken” FBA model. The biomass reaction is indicated by a bold line, reactions included in the model are indicated by solid lines, reactions omitted from the model (gaps) are indicated by dashed lines, and plausible exchange reactions omitted from the model are indicated by dotted/dashed lines.

Consider the reaction network depicted in Figure 5.  Table 1 contains the distance-to-biomass calculation. Metabolites *B*, *F*, *G*, *H*, and *I* will not be selected as having source reactions, and metabolites *A*, *B*, *C*, *D*, *E*, and *H* will not be selected as having escape reactions because they are infinite distance from the biomass reactants and products, respectively. The instance of (5) would be

(6) min⁡ xA+20xC+21xD+21xE+21yF+20yG+yI,s.t. −vA→E+bAsrc=0,   −vC→D+bCsrc=0,   −vDE→F+vC→D+bDsrc=0,   −vDE→F+vA→E+bEsrc=0,   −vF→GI+vDE→F−bFesc=0,    vF→GI−bGesc=0,    vF→GI−bIesc=0,    0≤bisrc≤Uisrcxi,   i∈{A,C,D,E},    0≤biesc≤Uiescyi, i∈{F,G,I},    L≤v≤U,

with the additional restriction that the variables *x*_*i*_ and *y*_*i*_ are binary. Included in the last set of constraints is a nonzero lower bound on *v*_*DE*→*F*_ which requires biomass production. The algorithm selects an artificial source reaction for metabolite *C* and an artificial escape for metabolite *G*. A source reaction for *C* is selected rather than a reaction for *D*, because *C* is further from the biomass reaction and therefore the cost is less. The selected source reactions will indicate to the modeler that reactions *B*↔*C* and *G*↔*H* are missing from the model. The reactions can be found hypothetically by searching through the database in MetModel GUI (Figure 3) or by searching the relevant pathway in another database. Adding these reactions and solving the new instance of (5) produces a solution that indicates that biologically plausible exchange reactions can be added for *A, B, I, *and *H* in order to produce biomass.

## 3. Results

**Application to a Partial Metabolic Reconstruction**

To illustrate the ability of FBA-Gap to aid in the construction of new FBA models, we apply the methodology to a new multicompartment model for *Cryptococcus neoformans*. *C. neoformans *is a fungus that can cause meningitis in humans. Because no metabolic reconstruction of *C. neoformans* has been previously carried out, we assign a generic biomass reaction previously used for *B. subtilis *[26] using only central metabolites that occur in the cytosol:

(7) 1.241 3 pg+2.097 AcCoa+1.236 Akg+35.115 ATP+0.397 e4p+0.428 g3p+0.712 g6p+0.542 Gly+14.405 NADPH+8.066 NH4+1.785 oaa+0.642 pep+1.640 Pi+2.994 Pyr+0.445 r5p+0.262 Ser-l+0.195 SO4→2.852 CO2+3.015 NADH.

We begin with a partial reconstruction based on evidence from genome annotations and scientific literature. The ability of the model to produce biomass is not considered during this step. The initial model consists of 576 reactions and 712 metabolites with compartments corresponding to the cytosol, mitochondria, and peroxisome. This initial curation was carried out over several weeks. We then solve (5) to find gaps in the model with a time limit of 120 seconds. The MetModel GUI database and KEGG are explored to find reactions that fill the gaps by producing and consuming metabolites with artificial exchange reactions. An internal reaction is added to the model only if it is present in the MetModel GUI database and if KEGG specifies that a gene encodes an enzyme that catalyzes the reaction in the organism. After adding reactions, (5) is solved again, and additional reactions are added. The process is repeated as long as plausible reactions can be added. 

**Figure 6:**
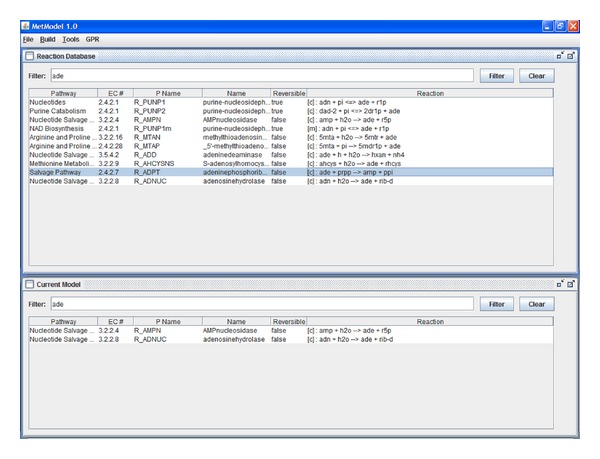
Searching for a gap-filling reaction is facilitated by a searchable and sortable database in MetModel GUI software. The initial model is unable to process ade^c^. The reaction database in MetModel GUI has 10 reactions that involve ade^e^. The KEGG database indicates that an enzyme for R_ADPT is encoded in the genome for *C. neoformans* and can be added to the model with confidence.

Four rounds of solving (5) and manually adding reactions from the MetModel GUI database/KEGG are conducted (Tables S4–S7).  Figure 6 illustrates how the searchable and sortable reaction database in MetModel GUI facilitates adding high-confidence reactions. After the first round of solving (5), cytoplasmic cyclic AMP is selected for an artificial source reaction. Browsing the MetModel GUI database reveals that the only reaction producing cyclic AMP, R_ADNCYC, is already included in the model. Upon inspection of the pathway containing R_ADNCYC, we discover an adjacent reaction, R_ADNK1, that is missing from the model and may be added because the corresponding enzyme is encoded in the *C. neoformans *genome. Cytoplasmic coenzyme A is also selected for an artificial source reaction. Inspection of the initial partial reconstruction reveals that much of fatty acid metabolism is omitted. Rather than adding the new pathways, we leave the artificial transport reaction as a placeholder. The artificial and plausible exchange reactions in Table S7 are sufficient to create a working model. In the final round of gap analysis, nine plausible source reactions, seven plausible escape reactions, and an artificial transport reaction for cytoplasmic coenzyme A are added to the model. The first round of solving (5) is terminated at the time limit of 120 seconds with a feasible solution. The remaining three rounds take less than one second to find a provably optimal solution. For a given metabolite, MetModel GUI instantly returns a list of potential reactions. For metabolites involved in reactions that form the backbone of the metabolic network, the list is typically short. Combined with searching the KEGG database, a round of FBA-Gap takes around ten minutes, and the completion of the partial reconstruction for *C. neoformans* takes around one hour.

**Comparison to Other Gap-Filling Algorithms**

In this section, FBA-Gap is compared to GapReed [8] and GapFind/GapFill [6] to demonstrate differences in results and computation time using the *C. neoformans *partial reconstruction. GapReed and GapFind/GapFill are implemented with a universal database curated from existing metabolic reconstructions.

When using GapFind/GapFill in these experiments, GapFind is applied to find all nonproducible metabolites in a model, and GapFill is applied to each nonproducible metabolite to find reactions to add so that the metabolite is produced. If GapFill is infeasible, an exchange reaction is added for that metabolite. Integer programming instances for all methods are solved using Gurobi (http://www.gurobi.com/) with a time limit of 600 seconds.

GapFind determines that only nine metabolites are producible and therefore there are 703 downstream unproducible metabolites. Solving the integer program for GapFind and the 703 integer programs for GapFill takes 50,169 seconds (about 14 hours). GapFill adds 550 reactions from the reaction database and 182 exchange reactions for metabolites. 

The integer program for GapReed terminates at the time limit of 600 seconds. Source exchange reactions are added for nine cytosolic metabolites, one escape exchange reaction is added, and one reaction from the reaction database is added. The source exchange reactions are for cytoplasmic Gln-L, SO_4_, FDP, O_2_, NADPH, Gly, PRPP, ATP, and Acetyl-CoA. The escape reaction is for cytoplasmic CO_2_, and the added reaction is the peroxidative reaction catalyzed by catalase (2H_2_O_2_ → 2H_2_O + O_2_).

GapFind/GapFill and GapReed are more conducive to an automated implementation than FBA-Gap, but in this example, one can see some of the pitfalls of an automated approach. GapFind/GapFill adds many internal reactions and exchange reactions for cytosolic metabolites so that there is a high probability that implausible reactions are present in the final model. Further, GapFind/GapFill requires significantly more computation time. GapReed adds exchange reactions for more implausible cytosolic metabolites than FBA-Gap. A hybrid computational/manual curation approach such as FBA-Gap is able to derive a biomass-producing model with higher-confidence reactions for our partial reconstruction for *C. neoformans* than these two established methods.

**Application to Existing Models**

FBA-Gap is applied to four existing models with exchange reactions removed. The metabolic reconstructions used in this experiment are for *Trypanosoma cruzi *[27]*, Bacillus subtilis* [26]*, Heliocbacter pylori *(iIT341 GSM/GPR) [2], and *Escherichia coli *(iJR904 GSM/GPR) [28]. The results of applying the procedure provide a hypothesis for a defined culture medium for each organism.

The hypothesized culture media are summarized in Table S2. *T. cruzi* is a protozoan parasite of humans that causes Chagas disease. The reconstruction is a multi-compartment model of central metabolism for *T. cruzi*. FBA-Gap selects biologically realistic source and escape reactions. The source reactions correspond to the transport of extracellular metabolites that are plausible constituents of a culture medium for sustaining *T. cruzi* and the escape reactions correspond to metabolites that are likely secreted by *T. cruzi. B. subtilis *is a Gram-positive bacterium found in soil. As with the *T. cruzi *model, FBA-Gap selects biologically realistic exchange reactions for production of biomass. For the *E. coli *reconstruction, one undesirable escape reaction is selected (clpn_ec^c^) that is unique to the *E. coli* model. This metabolite occurs in only one reaction in the model and is a reactant in the biomass reaction. Therefore, a simple remedy is to increase the stoichiometric coefficient in the biomass reaction. For the *h. pylori* reconstruction, a single implausible escape reaction is selected for rhcys^c^. Upon investigation of the network around this metabolite, we discover that there are reactions converting rhcys^c^ to dhptd^c^ and dhptd^c^ to hmfurn^c^, but there are no reactions consuming hmfurn^c^. There are no reactions consuming hmfurn^c^ in our universal database, so we can either add it as a reactant in the biomass reaction or add an escape reaction to remove it from the cell.

**Recovering Deleted Reactions from an Existing Model**

FBA-Gap is applied to an existing model with internal reactions deleted to evaluate the ability to find the resulting gaps in the network. In this experiment, we deleted a random sample of 222 internal reactions (15% of all reactions) from the *B. subtilis* model (Table S9). Solving (5) for the resulting model takes two seconds. FBA-Gap suggests 15 exchange reactions, all of which are source reactions (Table S10). Because we know which reactions are deleted from the model, we cannot properly evaluate how many of the deleted reactions a modeler would have added based on the suggested exchange reactions. Of the 15 metabolites in the selected artificial exchange reactions, 14 occur in at least one of the deleted reactions (93%), indicating that FBA-Gap does find the backbone metabolites that are next to the gaps. Of the 222 deleted reactions, 17 of them contain metabolites that are selected for artificial exchange reactions. Not all of the 222 deleted reactions are required to produce biomass, so it is likely that adding a subset of the deleted reactions would be sufficient for the resulting FBA model to produce biomass.

## 4. Discussion

This paper presents an optimization-based method for “debugging” metabolic reconstructions called FBA-Gap. We demonstrate the effectiveness of the procedure in helping to find gaps in a model for *C. neoformans. *FBA-Gap produces a more accurate reconstruction than an application of existing methods for filling gaps and requires less computation time. However, in contrast to other methods, FBA-Gap also involves manually selecting and approving which reactions to add to a model so that the overall time may be longer. As noted by Latendresse et al. [9], a fully automated gap-filling procedure likely leads to significant errors. The motivation behind FBA-Gap is to reduce the manual effort required by allowing the modeler to select from among a few suggested modifications to a model. The distance measure used in pricing artificial exchange reactions helps to indicate the location of gaps; these weights could also be incorporated into a procedure like MetaFlux [9], a more automated procedure that also has the capability of suggesting modifications to the biomass reaction. The FBA-Gap procedure provides hypotheses for defined culture media for organisms based on previously published models. One weakness of FBA-Gap is the computational complexity of solving (5). Finding optimal solutions to these integer programs is NP-Complete, but specialized solution methods may facilitate the computation of good solutions. 

## Supplementary Material

The Supplementary Material contains a proof that FBA-Gap is NP-Complete and tables related to computational results. A list of low-cost exchange reactions is in Table S1. Results from the first round of FBA-Gap for *B. subtilis*, *T. cruzi*, *H. pylori*, *E. coli*, and *C. neoformans* are in Table S2. For the *C. neoformans* model, the _rst step of FBA-Gap yields that the metabolites in Table S3 are an in_nite distance from the biomass reaction. The results of three rounds of gap analysis are in Tables S4-S7 below. Each table contains the transport reactions selected, and the action taken to avoid high-cost transports in the next round of gap analysis. In general, reactions are added only if they are in the MetModel GUI database and if KEGG specifies that a gene encodes the appropriate enzyme in the organism. The reactions in the final working model for *C. neoformans* is in Table S8. Table S9 contains reactions deleted from the B. subtilis model, and Table S10 contains the results of applying FBA-Gap to the broken model.

## Figures and Tables

**Table 1:** Distances of metabolites to biomass reactants and biomass products for the network depicted in Figure 5.

Distance to biomass reactants	Distance to biomass products
*d*_*A*_^src^ = 1	*d*_*A*_^esc^ = ∞
*d*_*B*_^src^ = ∞	*d*_*B*_^esc^ = ∞
*d*_*C*_^src^ = 1	*d*_*C*_^esc^ = ∞
*d*_*D*_^src^ = 0	*d*_*D*_^esc^ = ∞
*d*_*E*_^src^ = 0	*d*_*E*_^esc^ = ∞
*d*_*F*_^src^ = ∞	*d*_*F*_^esc^ = 0
*d*_*G*_^src^ = ∞	*d*_*G*_^esc^ = 1
*d*_*H*_^src^ = ∞	*d*_*H*_^esc^ = ∞
*d*_*I*_^src^ = ∞	*d*_*I*_^esc^ = 1

## List of Abbreviations

FBA: flux balance analysis
LP: linear program.
