# Supplementary material for: Gap Detection for Genome-Scale Constraint-Based Models
Source: Adv Bioinformatics. 2012 Sep 10;2012:323472. doi: 10.1155/2012/323472 (PMC3444828; doi:10.1155/2012/323472)
Supplement: Supplementary file 1 — The Supplementary Material contains a proof that FBA-Gap is NP-Complete and tables related to computational results. A list of low-cost exchange reactions is in Table S1. Results from the first round of FBA-Gap for B. subtilis, T. cruzi, H. pylori, E. coli, and C. neoformans are in Table S2. For the C. neoformans model, the _rst step of FBA-Gap yields that the metabolites in Table S3 are an in_nite distance from the biomass reaction. The results of three rounds of gap analysis are in Tables S4-S7 below. Each table contains the transport reactions selected, and the action taken to avoid high-cost transports in the next round of gap analysis. In general, reactions are added only if they are in the MetModel GUI database and if KEGG specifies that a gene encodes the appropriate enzyme in the organism. The reactions in the final working model for C. neoformans is in Table S8. Table S9 contains reactions deleted from the B. subtilis model, and Table S10 contains the results of applying FBA-Gap to the broken model. [file 323472.f1.pdf]

**Supplementary Material: Proof of NP-Completeness of FBA-GAP**

We specify an instance of the decision problem FBA-GAP as follows:

FBA-GAP[$R$, $M$, $k$, $l$, $\mathbf{L}$, $\mathbf{U}$, $\mathbf{L}^{src}, \mathbf{U}^{src}, \mathbf{L}^{esc}, \mathbf{U}^{esc}$]. Given a set of metabolites $M$ and reactions $R$, does there exist a set of $k$ source reactions and a set of $l$ escape reactions that can be added such that there exist fluxes satisfying the lower and upper bounds, including having flux through the biomass reaction more than $L_{biomass}$?

**Theorem.** *FBA-GAP is NP-Complete.*

*Proof.* Follows directly from Lemmas 1 and 2 below.

**Lemma 1.** *FBA-GAP is in NP.*

*Proof.* If a set of source reactions for a set of metabolites $M_s$ with $|M_s| = k$ and escape reactions for metabolites $M_e$ with $|M_e| = l$ is given, then set $x_i = 1$ for $i \in M_s$ and $x_i = 0$, otherwise and set $y_i = 1$ for $i \in M_e$ and $y_i = 0$, otherwise. Solve the following linear program

$$\max v_{biomass}$$

Subject to

$$
\begin{aligned}
&\mathbf{Sv} + \mathbf{b}^{esc} + \mathbf{b}^{esc} = \mathbf{0} \\
&\mathbf{L} \le \mathbf{v} \le \mathbf{U} \\
&(\mathbf{L}^{src})^T \mathbf{x} \le \mathbf{b}^{src} \le (\mathbf{U}^{src})^T \mathbf{x} \\
&(\mathbf{L}^{esc})^T \le \mathbf{b}^{esc} \le (\mathbf{U}^{esc})^T \mathbf{y}
\end{aligned}
$$

If the objective value associated with an optimal solution has $v_{biomass} \ge L_{biomass}$, then the sets of source and escape reactions are sufficient. Solving the linear program is polynomial in the size of the inputs, so FBA-GAP is in *NP*.

**Lemma 2.** *FBA-GAP is NP-hard.*

*Proof.* CLOSED HEMISPHERE, known to be strongly NP-complete (Johnson & Preparata, 1978), can be reduced to FBA-GAP. The decision problem for CLOSED HEMISPHERE is stated as follows:

CLOSED HEMISPHERE [*m*, *d*, ***A***, *n*]. Given a set of *m* linear inequalities $\boldsymbol{Ax} \ge 0 : \boldsymbol{A} \in \mathbb{R}^{m \times d}$ does there exist an $x \in \mathbb{R}^d$ such that $\| x \| > 0$ and at least *n* of the inequalities are satisfied?

Suppose an instance of CLOSED HEMISPHERE, [*m*, *d*, ***A***, *n*] is given. The problem is equivalent to the following intermediate problem: given *m*, *d*, ***A***, is there an $x \in \mathbb{R}^d$ with $\| \boldsymbol{x} \| > 0$ and an $\boldsymbol{s} \in \mathbb{R}_+^m$ such that at least *n* of the equalities $\boldsymbol{Ax} - \boldsymbol{s} = 0$ is satisfied?

This intermediate problem can be phrased as a collection of $d$ instances of FBA-GAP. Each column of $\boldsymbol{A}$ corresponds to a reaction and each row corresponds to a metabolite. Positive elements in a column of $\boldsymbol{A}$ are the stoichiometric coefficients for products and negative elements are (the negative of) stoichiometric coefficients for reactants. Each variable $x_j$ corresponds to the flux through reaction $j$. For now, there are no bounds on the flux through each reaction. Each of the $s_i$ variables corresponds to a one-sided escape reaction for a metabolite.

With the sets of metabolites $M$ and reactions $R$ as specified, create an instance of FBA-GAP for each $x_j, j = 1,\ldots,d$ where $x_j$ corresponds to the flux through the biomass reaction $v_{biomass}$. Set $L_{biomass} = 0$, for each instance. Each instance of FBA-GAP involves determining if there exists a set of $k = m - n$ source reactions (and $l = 0$ escape reactions) such that the flux through the biomass reaction is more than 0.

If the answer to an instance of CLOSED HEMISPHERE is 'yes', then there exists a certificate for the instance with $x_j > 0$ for some $j$. This certificate will solve the instance of FBA-GAP where $x_j$ corresponds to $v_{biomass}$. If one of the $d$ instances of FBA-GAP has a 'yes' certificate, then there is a solution to the intermediate problem with the corresponding $x_j > 0$. Therefore, $\| \boldsymbol{x} \| > 0$, and the instance of CLOSED HEMISPHERE has a 'yes' certificate. The transformation of CLOSED HEMISPHERE to $d$ instances of FBA-GAP involves only the

introduction of the $s_i$ variables for each instance, and is therefore a polynomial-time transformation.
