# Supplementary material for: Gap Detection for Genome-Scale Constraint-Based Models
Source: Adv Bioinformatics. 2012 Sep 10;2012:323472. doi: 10.1155/2012/323472 (PMC3444828; doi:10.1155/2012/323472)
Supplement: Supplementary file 2 [file 323472.f2.pdf]

Supplementary Material

March 6, 2012

List of Tables

S8 Reactions in Final Working Model for *C. neoformans* 2

S1 Low-Cost Exchange Reactions 22

S2 Results of First Round of FBA-Gap 23

S3 Metabolites that are distance ∞ from biomass reaction for *C. neoformans* 24

S4 Round 1 of Gap Analysis for *C. neoformans* 25

S5 Round 2 of Gap Analysis for *C. neoformans* 25

S6 Round 3 of Gap Analysis for *C. neoformans* 26

S7 Round 4 of Gap Analysis for *C. neoformans* 26

Results from the first round of FBA-Gap for *B. subtilis*, *T. cruzi*, *H. pylori*, *E. coli*, and *C. neoformans* are in Table S2.

For the *C. neoformans* model, the first step of FBA-Gap yields that the metabolites in Table S3 are an infinite distance from the biomass reaction. The results of three rounds of gap analysis are in Tables S4-S6 below. Each table contains the transport reactions selected, and the action taken to avoid high-cost transports in the next round of gap analysis. In general, reactions are added only if they are in the MetModel GUI database and if KEGG specifies that a gene encodes the appropriate enzyme in the organism. The reactions in the final working model for *C. neoformans* is in Table S8.

Table S8: Reactions in Final Working Model for *C. neoformans*

Pathway	EC #	Reaction Code	Reversible	Reaction Name	Reaction
–	.	R_HCO3En	true	HCO3equilibriumreaction, nuclear	[n] : co2 + h2o <=> h + hco3
Alanine and Aspartate Metabolism	2.1.1.10	R_HCYSMT	false	homocysteineS-methyl-transferase	[c] : amet + hcys_l → ahcys + h + met_l
Alanine and Aspartate Metabolism	2.3.1.7	R_CSNATirm	false	carnitineO-acetyl-transferase, reversedirection, mitochondrial	[m] : accoa + crn → acrn + coa
Alanine and Aspartate Metabolism	2.3.1.7	R_CSNATifm	false	carnitineO-aceyl-transferase, forwardreaction, mitochondrial	[m] : acrn + coa → accoa + crn
Alanine and Aspartate Metabolism	6.1.1.12	R_ASPTRS	false	Aspartyl-tRNAsynthetase	[c] : asp_l + atp + trnaasp → amp + asptrna + ppi
Alanine and Aspartate Metabolism	6.1.1.12	R_ASPTRSm	false	Aspartyl-tRNAsynthetase, mitochondrial	[m] : asp_l + atp + trnaasp → amp + asptrna + ppi
Alanine and Aspartate Metabolism	2.6.1.1	R_ASPTAp	true	aspartatetransaminase, peroxisomal	[x] : akg + asp_l <=> glu_l + oaa
Alanine and Aspartate Metabolism	2.6.1.1	R_ASPTA	true	aspartatetransaminase	[c] : akg + asp_l <=> glu_l + oaa
Alanine and Aspartate Metabolism	2.6.1.1	R_ASPTAm	true	aspartatetransaminase	[m] : akg + asp_l <=> glu_l + oaa
Alanine and Aspartate Metabolism	2.7.2.4	R_ASPKi	false	aspartatekinase, irreversible	[c] : asp_l + atp → 4pasp + adp
Alanine and Aspartate Metabolism	6.3.5.4	R_ASNS1	false	asparaginesynthase (glutamine-hydrolysing)	[c] : asp_l + atp + gln_l + h2o → amp + asn_l + glu_l + h + ppi
Alanine and Aspartate Metabolism	1.2.1.11	R_ASADi	false	aspartate-semialdehydedehydrogenase, irreversible	[c] : 4pasp + h + nadph → aspsa + nadp + pi
Alanine and Aspartate Metabolism	6.1.1.7	R_ALATRS	false	Alanyl-tRNAsynthetase	[c] : ala_l + atp + trnaala → alatrna + amp + ppi
Alanine and Aspartate Metabolism	2.6.1.2	R_ALATA_Lm	true	L-alaninetransaminase, mitochondrial	[m] : akg + ala_l <=> glu_l + pyr
Glutamate metabolism	2.6.1.2	R_ALATA_L	true	L-alaninetransaminase	[c] : akg + ala_l <=> glu_l + pyr
Starch and Sucrose Metabolism	3.2.1.28	R_TREH	false	alpha, alpha-trehalase	[c] : h2o + tre → 2.0 glc_d
Alternate Carbon Metabolism	2.4.1.15	R_TRE6PS	false	alpha, alpha-trehalose--phosphatesynthase (UDP-forming)	[c] : g6p + udpg → h + tre6p + udp
Alternate Carbon Metabolism	2.4.1.15	R_TRE6PP	false	trehalose-phosphatase	[c] : h2o + tre6p → pi + tre
Galactose metabolism	3.2.1.26	R_SUCRe	false	sucrosehydrolyzingenxyme, extracellular	[e] : h2o + sucr → fru + glc_d
Fructose and Mannose Metabolism	1.1.1.2	R_SBTR	false	D-sorbitolreductase	[c] : glc_d + h + nadph → nadp + sbt_d
Alternate Carbon Metabolism	1.1.1.14	R_SBTD_L	false	L-sorbitoldehydrogenase (L-sorbose-producing)	[c] : nad + sbt_l → h + nadh + srb_l
Fructose and Mannose Metabolism	1.1.1.14	R_SBTD_D2	false	D-sorbitoldehydrogenase (D-fructoseproducing)	[c] : nad + sbt_d → fru + h + nadh
Pyruvate Metabolism	4.4.1.5	R_LGTHL	false	lactoylglutathionelyase	[c] : gthrd + mthgxl → lgt_s
Alternate Carbon Metabolism	1.1.2.3	R_L_DASH_LACDm	false	L-Lactatedehydrogenase, mitochondrial	[m] : 2.0 ficytc + lac_l → focytc + pyr
Pyruvate Metabolism	3.1.2.6	R_GLYOXm	false	hydroxyacylglutathionehydrolase, mitochondrial	[m] : h2o + lgt_s → gthrd + h + lac_d
Pyruvate Metabolism	3.1.2.6	R_GLYOX	false	hydroxyacylglutathionehydrolase	[c] : h2o + lgt_s → gthrd + h + lac_d
Alternate Carbon Metabolism	2.4.1.11	R_GLYGS	false	glycogen (starch)synthase	[c] : h2o + udpg → 14glun + h + udp
Alternate Carbon Metabolism	2.4.1.11	R_GLCS2	false	glycogensynthase (UDPGlc)	[c] : udpg → glycogen + h + udp
Alternate Carbon Metabolism	2.4.1.1	R_GLCP	false	glycogenphosphorylase	[c] : glycogen + pi → g1p
Alternate Carbon Metabolism	2.4.1.18	R_GBEZ	false	_1, 4-alpha-glucanbranchingenzyme	[c] : 14glun → glycogen + h2o

Alternate Carbon Metabolism	1.1.2.4	R_D_DASH_LACDm	false	D-lactatedehydrogenase, mitochondrial	[m] : 2.0 ficytc + lac_d → focytc + pyr
Alternate Carbon Metabolism	2.4.1.34	R_13GS	false	_1, 3-beta-glucansynthase	[c] : udpg → 13bdglcn + h + udp
Aminosugar Metabolism	3.5.1.41	R_CHTNDA	false	chitindeacetylase	[c] : chitin + h2o → ac + chitos + h
Anaplerotic reactions	4.1.1.49	R_PPCK	false	phosphoenolpyruvate-carboxykinase	[c] : atp + oaa → adp + co2 + pep
Anaplerotic reactions	6.4.1.1	R_PC	false	pyruvatecarboxylase	[c] : atp + hco3 + pyr → adp + h + oaa + pi
Pyruvate Metabolism	1.1.1.40	R_ME2m	false	malicenzyme (NADP), mitochondrial	[m] : mal_l + nadp → co2 + nadph + pyr
Pyruvate Metabolism	1.1.1.38	R_ME1m	false	malicenzyme (NAD), mitochondrial	[m] : mal_l + nad → co2 + nadh + pyr
Anaplerotic reactions	4.1.3.2	R_MALSp	false	malatesynthase	[x] : accoa + glx + h2o → coa + h + mal_l
Glycolysis/Gluconeogenesis	2.7.1.11	R_FBP	false	fructose-bisphosphatase	[c] : fdp + h2o → f6p + pi
Pentose and Glucuronate Interconversions	1.1.1.2	R_ARABR	false	arabinosereductase	[c] : arab_l + h + nadph → abt + nadp
Arabinose Metabolism	1.1.1.116	R_ARAB1D2	false	D-arabinose1-dehydrogenase (NADP)	[c] : arab_d + nadp → dara14lac + h + nadph
Arabinose Metabolism	1.1.1.116	R_ARAB1D1	false	D-arabinose1-dehydrogenase (NAD)	[c] : arab_d + nad → dara14lac + h + nadh
Urea cycle/amino group metabolism	2.5.1.16	R_SPMS	false	spermidinesynthase	[c] : ametam + ptrc → 5mta + h + spmd
Arginine and Proline Metabolism	6.1.1.15	R_PROTRS	false	Prolyl-tRNAsynthetase	[c] : atp + pro_l + trnapro → amp + ppi + protrna
Arginine and Proline Metabolism	.	R_PRO1xm	false	prolineoxidase (NAD), mitochondrial	[m] : nad + pro_l → 1pyr5c + h + nadh
Arginine and Proline Metabolism	1.5.1.12	R_PHCDm	false	L-1-pyrroline-3-hydroxy-5-carboxylatedehydrogenase	[m] : 1p3h5c + 2.0 h2o + nad → e4hglu + h + nadh
Urea cycle/amino group metabolism	1.5.1.2	R_P5CR	false	pyrroline-5-carboxylate-reductase	[c] : 1pyr5c + 2.0 h + nadph → nadp + pro_l
Arginine and Proline Metabolism	2.3.1.35	R_ORNTACim	false	ornithinetransacetylase, irreversible, mitochondrial	[m] : acorn + glu_l → acglu + orn
Arginine and Proline Metabolism	2.6.1.13	R_ORNTA	false	ornithinetransaminase	[c] : akg + orn → glu_l + glu5sa
Urea cycle/amino group metabolism	4.1.1.17	R_ORNDC	false	OrnithineDecarboxylase	[c] : h + orn → co2 + ptrc
Arginine and Proline Metabolism	2.1.3.3	R_OCBTi	false	ornithinecarbamoyl-transferase, irreversible	[c] : cbp + orn → citr_l + h + pi
Arginine and Proline Metabolism	1.5.1.12	R_HPROym	false	L-hydroxyprolinedehydrogenase (NADP), mitochondrial	[m] : 4hpro_lt + nadp → 1p3h5c + h + nadph
Arginine and Proline Metabolism	1.5.1.12	R_HPROxm	false	L-hydroxyprolinedehydrogenase (NAD), mitochondrial	[m] : 4hpro_lt + nad → 1p3h5c + h + nadh
Arginine and Proline Metabolism	1.5.1.2	R_HPROa	false	L-hydroxyproline-reductase (NAD)	[c] : 1p3h5c + 2.0 h + nadh → 4hpro_lt + nad
Arginine and Proline Metabolism	1.5.1.2	R_HPROb	false	L-hydroxyproline-reductase (NADP)	[c] : 1p3h5c + 2.0 h + nadph → 4hpro_lt + nadp
Arginine and Proline Metabolism	2.7.2.11	R_GLU5K	false	glutamate5-kinase	[c] : atp + glu_l → adp + glu5p
Arginine and Proline Metabolism	1.2.1.41	R_G5SD	false	glutamate-5-semialdehydedehydrogenase	[c] : glu5p + h + nadph → glu5sa + nadp + pi
Arginine and Proline Metabolism	1.2.1.41	R_G5SD2	false	glutamate-5-semialdehydedehydrogenase	[c] : glu5p + h + nadh → glu5sa + nad + pi
Arginine and Proline Metabolism	.	R_G5SADr	true	L-glutamate5-semialdehydedehydratase, reversible	[c] : glu5sa <=> 1pyr5c + h + h2o
Arginine and Proline Metabolism	.	R_G5SADrm	true	L-glutamate5-semialdehydedehydratase, reversible, mitochondrial	[m] : glu5sa <=> 1pyr5c + h + h2o
Arginine and Proline Metabolism	2.6.1.1	R_EHGLATp	false	L-erythro-4-Hydroxyglutamate:2-oxoglutarateamino-transferase, peroxisomal	[x] : akg + e4hglu → 4h2oglt + glu_l

Arginine and Proline Metabolism	2.6.1.1	R_EHGLAT	false	L-erythro-4-Hydroxyglutamate:2-oxoglutarateamino-transferase	[c] : akg + e4hglu → 4h2oglt + glu_l
Arginine and Proline Metabolism	2.6.1.1	R_EHGLATm	false	L-erythro-4-Hydroxyglutamate:2-oxoglutarateamino-transferase, mitochondrial	[m] : akg + e4hglu → 4h2oglt + glu_l
Pyrimidine Biosynthesis	3.5.2.3	R_CBPS	false	carbamoyl-phosphate-synthase (glutamine-hydrolysing)	[c] : 2.0 atp + gln_l + h2o + hco3 → adp + cbp + glu_l + h + pi
Arginine and Proline Metabolism	6.3.5.5	R_CBPSn	false	carbamoyl-phosphate-synthase (glutamine-hydrolysing), nuclear	[n] : 2.0 atp + gln_l + h2o + hco3 → adp + cbp + glu_l + h + pi
Arginine and Proline Metabolism	6.1.1.19	R_ARGTRS	false	Arginyl-tRNAsynthetase	[c] : arg_l + atp + trnaarg → amp + argtrna + ppi
Arginine and Proline Metabolism	6.1.1.19	R_ARGTRSm	false	Arginyl-tRNAsynthetase, mitochondrial	[m] : arg_l + atp + trnaarg → amp + argtrna + ppi
Arginine and Proline Metabolism	6.3.4.5	R_ARGSSr	true	argininosuccinatesynthase, reversible	[c] : asp_l + atp + citr_l <=> amp + argsuc + h + ppi
Alanine and Aspartate Metabolism	4.3.2.1	R_ARGSL	true	argininosuccinatelyase	[c] : argsuc <=> arg_l + fum
Urea cycle/amino group metabolism	3.5.3.1	R_ARGN	false	arginase	[c] : arg_l + h2o → orn + urea
Arginine and Proline Metabolism	3.5.1.4	R_AMID	false	amidase	[c] : 4gudbd + h2o → 4gudbutn + nh4
Urea cycle/amino group metabolism	1.2.1.38	R_AGPRim	false	N-acetyl-g-glutamyl--phosphatereductase, irreversible, mitochondrial	[m] : acg5p + h + nadph → acg5sa + nadp + pi
Arginine and Proline Metabolism	4.1.1.50	R_ADMDC	false	adenosylmethioninedecarboxylase	[c] : amet + h → ametam + co2
Arginine and Proline Metabolism	2.6.1.11	R_ACOTAim	false	acteylornithinetransaminase irreversible, mitochondrial	[m] : acg5sa + glu_l → acorn + akg
Urea cycle/amino group metabolism	2.3.1.1	R_ACGSm	false	N-acteylglutamatesynthase, mitochondrial	[m] : accoa + glu_l → acglu + coa + h
Arginine and Proline Metabolism	1.2.1.38	R_ACGKm	false	acetylglutamatekinase, mitochondrial	[m] : acglu + atp → acg5p + adp
Arginine and Proline Metabolism	1.5.1.12	R_4HGLSDm	true	L-4-hydroxyglutamatesemialdehydedehydrogenase, mitochondrial	[m] : 4hglusa + h2o + nad <=> e4hglu + h + nadh
Asparagine metabolism	6.1.1.22	R_ASNTRSm	false	asparaginyl-tRNAsynthetase, miotchondrial	[m] : asn_l + atp + trnaasn → amp + asntrna + ppi
Asparagine metabolism	6.1.1.22	R_ASNTRS	false	Asparaginyl-tRNAsynthetase	[c] : asn_l + atp + trnaasn → amp + asntrna + ppi
Asparagine metabolism	3.5.1.1	R_ASNN	false	L-asparaginase	[c] : asn_l + h2o → asp_l + nh4
Asparagine metabolism	3.5.1.1	R_ASNNe	false	L-asparaginase, extracellular	[e] : asn_l + h2o → asp_l + nh4
Citric Acid Cycle	6.2.1.4	R_SUCOAS1m	true	Succinate–CoAligase (GDP-forming)	[m] : coa + gtp + succ <=> gdp + pi + succoa
Citric Acid Cycle	1.3.5.1	R_SUCD2_u6m	true	succinatedehydrogenase (ubiquinone-6), mitochondrial	[m] : q6 + succ <=> fum + q6h2
Citric Acid Cycle	1.1.1.42	R_ICDHyp	false	Isocitratedehydrogenase (NADP+)	[x] : icit + nadp → akg + co2 + nadph
Citric Acid Cycle	1.1.1.42	R_ICDHym	false	Isocitratedehydrogenase (NADP+)	[m] : icit + nadp → akg + co2 + nadph
Citric Acid Cycle	1.1.1.42	R_ICDHy	false	isocitratedehydrogenase (NADP)	[c] : icit + nadp → akg + co2 + nadph
Citric Acid Cycle	1.1.1.41	R_ICDHxm	false	Isocitratedehydrogenase (NAD+)	[m] : icit + nad → akg + co2 + nadh
Citric Acid Cycle	4.1.3.7	R_CSp	false	citratesynthase	[x] : accoa + h2o + oaa → cit + coa + h
Citric Acid Cycle	4.1.3.7	R_CSm	false	citratesynthase	[m] : accoa + h2o + oaa → cit + coa + h
Citric Acid Cycle	1.2.4.2	R_AKGDbm	false	oxoglutaratedehydrogenase (dihydrolipoamideS-succinyltransferase)	[m] : coa + sdhlam → dhlam + succoa
Citric Acid Cycle	1.2.4.2	R_AKGDam	true	oxoglutaratedehydrogenase (lipoamide)	[m] : akg + h + lpam <=> co2 + sdhlam
Citric Acid Cycle	4.2.1.3	R_ACONT	true	aconitase	[c] : cit <=> icit
Citric Acid Cycle	4.2.1.3	R_ACONTm	true	Aconitatehydratase	[m] : cit <=> icit

Cysteine Metabolism	2.3.1.30	R_SERATi	false	serineO-acetyl-transferase, irreversible	[c] : accoa + ser_l → acser + coa
Nucleotides	2.7.1.25	R_SADT	false	sulfateadenylyl-transferase	[c] : atp + h + so4 → aps + ppi
Cysteine Metabolism	6.1.1.16	R_CYSTRS	false	Cysteinyl-tRNAsynthetase	[c] : atp + cys_l + trnacys → amp + cystrna + ppi
Cysteine Metabolism	2.5.1.47	R_CYSS	false	cysteinesynthase	[c] : acser + h2s → ac + cys_l + h
Nucleotides	3.1.3.7	R_BPNT	false	_3', 5'-bisphosphate-nucleotidase	[c] : h2o + pap → amp + pi
Nucleotides	2.7.1.25	R_ADSK	false	adenylyl-sulfatekinase	[c] : aps + atp → adp + h + paps
Fatty Acid Biosynthesis	2.7.7.1	R_NMNAT	false	nicotinamide--nucleotideadenylyl-transferase	[c] : atp + h + nmn → nad + ppi
Fatty Acid Biosynthesis	3.6.1.22	R_NADDPp	false	NADdiphosphatase, peroxisomal	[x] : h2o + nad → amp + h + nmn
Fatty Acid Biosynthesis	2.3.1.16	R_KATp	false	_3-ketoacyl-CoAthiolase, peroxisomal	[x] : 3oacoa + coa → accoa + acoa
Fatty Acid Biosynthesis	1.1.1.35	R_HACDp	true	_3-hydroxyacyl-CoAdehydrogenase	[x] : 3hacoa + nad <=> 3oacoa + h + nadh
Folate Metabolism	6.3.2.17	R_THFGLUS	true	Tetrahydrofolate:L-glutamategamma-ligase (ADP-forming)	[c] : atp + glu_l + thf <=> adp + h + pi + thfglu
Folate Metabolism	1.2.4.1	R_THFATm	false	tetrahydrofolateamino-methyltransferase, mitochondrial	[m] : h2o + methf → 5fthf + h
Tetrahydrobiopterin	3.5.4.16	R_GTPCI	false	GTPcyclo-hydrolaseI	[c] : gtp + h2o → ahdt + for + h
Folate Metabolism	2.1.2.9	R_FMETTRSm	false	Methionyl-tRNAformyl-transferase, mitochondrial	[m] : 10fthf + mettrna → fmettrna + h + thf
Folate Metabolism	3.6.3.16	R_DNMPPA	false	Dihydroneopterinmonophosphatedephosphorylase	[c] : dhpmp + h2o → dhnpt + pi
Folate Metabolism	4.1.3.-	R_ADCS	false	_4-amino-4-deoxychorismatesynthase	[c] : chor + gln_l → 4adcho + glu_l
Fructose and Mannose Metabolism	5.4.2.2	R_PMANM	true	phosphomannomutase	[c] : man1p <=> man6p
Fructose and Mannose Metabolism	2.7.1.105	R_PFK26	false	_6-phosphofructo-2-kinase	[c] : atp + f6p → adp + f26bp + h
Fructose and Mannose Metabolism	5.3.1.8	R_MAN6PI	true	mannose-6-phosphate-isomerase	[c] : man6p <=> f6p
Fructose and Mannose Metabolism	2.7.7.13	R_MAN1PT	false	mannose-1-phosphate-guanylyltransferase	[c] : gtp + h + man1p → gdpmann + ppi
Fructose and Mannose Metabolism	2.7.1.105	R_FBP26	false	Fructose-2, 6-bisphosphate2-phosphatase	[c] : f26bp + h2o → f6p + pi
Galactose metabolism	2.7.7.12	R_UGLT	true	UDPglucose–hexose-1-phosphateuridylyl-transferase	[c] : gal1p + udpg <=> g1p + udpgal
Galactose metabolism	5.1.3.2	R_UDPG4E	true	UDPglucose4-epimerase	[c] : udpg <=> udpgal
Starch and Sucrose Metabolism	3.2.1.20	R_MALT	false	alpha-glucosidase	[c] : h2o + malt → 2.0 glc_d
Galactose metabolism	2.7.7.9	R_GALU	true	UTP-glucose-1--phosphateuridylyl-transferase	[c] : g1p + h + utp <=> ppi + udpg
Galactose metabolism	2.7.7.10	R_GALT	true	galactose-1-phosphate-uridylyltransferase	[c] : gal1p + h + utp <=> ppi + udpgal
Galactose metabolism	2.7.1.6	R_GALK	false	galactokinase	[c] : atp + gal → adp + gal1p + h
Galactose metabolism	3.2.1.20	R_DGGH	false	alpha-D-glucosideglucohydrase	[c] : 6dg + h2o → gal + glc_d
Glutamate metabolism	2.7.7.23	R_UDPACGLP	true	UDP-N-acetylglucosaminediphosphorylase	[c] : acgam1p + h + utp <=> ppi + udpacgal
Glutamate metabolism	1.2.1.16	R_SSALy	false	succinate-semialdehydedehydrogenase (NADP)	[c] : h2o + nadp + sucsal → h + nadph + succ
Glutamate metabolism	1.5.1.12	R_PYR5CDm	false	D1-pyrroline-5-carboxylatedehydrogenase, mitochondrial	[m] : glu5sa + h2o + nadp → glu_l + h + nadph
Glutamate metabolism	1.4.1.13	R_GLUSx	false	glutamatesynthase (NADH2)	[c] : akg + gln_l + h + nadh → glu_l + nad
Glutamate metabolism	1.4.1.4	R_GLUDy	true	glutamatedehydrogenase (NADP)	[c] : glu_l + h2o + nadp <=> akg + h + nadph + nh4
Glutamate metabolism	4.1.1.15	R_GLUDC	false	GlutamateDecarboxylase	[c] : glu_l + h → 4abut + co2

Glutamate metabolism	1.4.1.2	R_GLUDxi	false	glutamatede-hydrogenase (NAD)	[c] : glu_l + h2o + nad → akg + h + nadh + nh4
Glutamate metabolism	2.3.1.7	R_CSNATirp	false	carnitineO-acetyl-transferase, reversedi-rection, peroxisomal	[x] : accoa + crn → acrn + coa
Glutamate metabolism	2.4.1.16	R_CHTNS	false	chitinsynthase	[c] : udpacgal → chitin + h + udp
Glutamate metabolism	2.6.1.19	R_ABTA	false	_4-aminobutyratetransaminase	[c] : 4abut + akg → glu_l + sucsal
Glutamine Metabolism	6.1.1.17	R_GLUTRSm	false	glutamyl-tRNAsynthetase, mitochondrial	[m] : atp + glu_l + trnaglu → amp + glutrna + ppi
Glutamine Metabolism	6.1.1.17	R_GLUTRS	false	Glutamyl-tRNAsynthetase	[c] : atp + glu_l + trnaglu → amp + glutrna + ppi
Glutamine Metabolism	3.5.1.2	R_GLUN	false	glutaminase	[c] : gln_l + h2o → glu_l + nh4
Glutamine Metabolism	6.1.1.18	R_GLNTRS	false	Glutaminyl-tRNAsynthetase	[c] : atp + gln_l + trnagln → amp + glntrna + ppi
Glutamate metabolism	6.3.1.2	R_GLNS	false	glutaminesynthetase	[c] : atp + glu_l + nh4 → adp + gln_l + h + pi
Aminosugar Metabolism	2.6.1.16	R_GF6PTA	false	glutamine-fructose-6--phosphatetransaminase	[c] : f6p + gln_l → gam6p + glu_l
Glutamine Metabolism	4.1.3.27	R_ANS	false	anthranilatesynthase	[c] : chor + gln_l → anth + glu_l + h + pyr
Glycine and Serine Metabolism	6.1.1.11	R_SERTRS	false	Seryl-tRNAsynthetase	[c] : atp + ser_l + trnaser → amp + ppi + sertrna
Glycine and Serine Metabolism	4.2.1.13	R_SERD_L	false	L-serinedeaminase	[c] : ser_l → nh4 + pyr
Glycine, Serine, and Threonine Metabolism	3.1.3.3	R_PSP_L	false	phospho-serinephosphatase (L-serine)	[c] : h2o + pser_l → pi + ser_l
Glycine, Serine, and Threonine Metabolism	2.6.1.52	R_PSERT	false	phospho-serinetransaminase	[c] : 3php + glu_l → akg + pser_l
Glycine, Serine, and Threonine Metabolism	1.1.1.95	R_PGCD	false	phosphoglyceratede-hydrogenase	[c] : 3pg + nad → 3php + h + nadh
Glycine and Serine Metabolism	2.7.1.39	R_HSK	false	homoserinekinase	[c] : atp + hom_l → adp + h + phom
Glycine and Serine Metabolism	1.1.1.3	R_HSDxi	false	homoserinede-hydrogenase (NADH), irreversible	[c] : aspsa + h + nadh → hom_l + nad
Glycine and Serine Metabolism	1.1.1.3	R_HSDyi	false	homoserinede-hydrogenase (NADP), irreversible	[c] : aspsa + h + nadph → hom_l + nadp
Glycine and Serine Metabolism	6.1.1.14	R_GLYTRS	false	Glycyl-tRNAsynthetase	[c] : atp + gly + trnagly → amp + glytrna + ppi
Glycine and Serine Metabolism	1.4.4.2	R_GLYCLm	false	glycinecleavagesystem, mitochondrial	[m] : gly + nad + thf → co2 + mlthf + nadh + nh4
Glycine, Serine, and Threonine Metabolism	2.1.2.1	R_GHMT2rm	true	glycinehydroxymethyl-transferase, reversible, mitochondrial	[m] : ser_l + thf <=> gly + h2o + mlthf
Glycine, Serine, and Threonine Metabolism	2.1.2.1	R_GHMT2r	true	glycinehydroxymethyl-transferase, reversible	[c] : ser_l + thf <=> gly + h2o + mlthf
Glycine, Serine, and Threonine Metabolism	1.4.4.2	R_GCCcm	true	glycine-cleavagecomplex (lipoylprotein), mito-chondrial	[m] : dhlpro + nad <=> h + lpro + nadh
Glycine, Serine, and Threonine Metabolism	1.4.4.2	R_GCCbim	false	glycine-cleavagecomplex (lipoylprotein)ir-reversible, mitochon-drial	[m] : alpro + thf → dhlpro + mlthf + nh4
Glycine, Serine, and Threonine Metabolism	1.4.4.2	R_GCCam	true	glycine-cleavagecomplex (lipoylprotein), mito-chondrial	[m] : gly + h + lpro <=> alpro + co2
Glycine, Serine, and Threonine Metabolism	1.4.4.2	R_GCC2cm	true	glycine-cleavagecomplex (lipoamide), mitochon-drial	[m] : dhlam + nad <=> h + lpam + nadh
Glycine, Serine, and Threonine Metabolism	1.4.4.2	R_GCC2bim	false	glycine-cleavagesystem (lipoamide)irreversible, mitochondrial	[m] : alpam + thf → dhlam + mlthf + nh4
Glycine, Serine, and Threonine Metabolism	1.4.4.2	R_GCC2am	true	glycine-cleavagecomplex (lipoamide), mitochon-drial	[m] : gly + h + lpam <=> alpam + co2
Methionine Metabolism	4.2.1.22	R_CYSTS	false	cystathioninebeta-synthase	[c] : hcys_l + ser_l → cyst_l + h2o

Glycine and Serine Metabolism	2.6.1.-	R_AGT	true	alanine-glyoxylatetransaminase	[c] : ala_l + glx <=> gly + pyr
Glycolysis/Gluconeogenesis	5.3.1.1	R_TPI	true	triose-phosphate-isomerase	[c] : dhap <=> g3p
Glycolysis/Gluconeogenesis	2.7.1.40	R_PYK	false	pyruvatekinase	[c] : adp + h + pep → atp + pyr
Glycolysis/Gluconeogenesis	5.4.2.1	R_PGM	true	phospho-glyceratemutase	[c] : 2pg <=> 3pg
Glycolysis/Gluconeogenesis	2.7.2.3	R_PGK	true	phosphoglyceratekinase	[c] : 3pg + atp <=> 13dpg + adp
Glycolysis/Gluconeogenesis	5.3.1.9	R_PGI	true	glucose-6-phosphate-isomerase	[c] : g6p <=> f6p
Glycolysis/Gluconeogenesis	1.2.4.1	R_PDHm	false	pyruvatedehydrogenase	[m] : coa + nad + pyr → accoa + co2 + nadh
Glycolysis/Gluconeogenesis	1.2.4.2	R_PDHcm	false	pyruvatedehydrogenase (dihydrolipoamidedehydrogenase)	[m] : dhlam + nad → h + lpam + nadh
Fructose and Mannose Metabolism	2.7.1.1	R_HEX7	false	hexokinase (D-fructose:ATP)	[c] : atp + fru → adp + f6p + h
Fructose and Mannose Metabolism	2.7.1.1	R_HEX4	false	hexokinase (D-mannose:ATP)	[c] : atp + man → adp + h + man6p
Glycolysis/Gluconeogenesis	2.7.1.1	R_HEX1	false	hexokinase (D-glucose:ATP)	[c] : atp + glc_d → adp + g6p + h
Glycolysis/Gluconeogenesis	2.7.1.2	R_GLUK	false	Glucokinase	[c] : atp + glc_d → adp + g6p_b + h
Glycolysis/Gluconeogenesis	1.2.1.12	R_GAPD	true	glyceraldehyde-3-phosphatedehydrogenase	[c] : g3p + nad + pi <=> 13dpg + h + nadh
Glycolysis/Gluconeogenesis	5.3.1.9	R_G6PI2	true	Glucose-6-phosphate-isomerase	[c] : g6p_b <=> f6p_b
Glycolysis/Gluconeogenesis	5.3.1.9	R_G6PI	true	Glucose-6-phosphate-isomerase	[c] : g6p <=> g6p_b
Glycolysis/Gluconeogenesis	4.1.2.13	R_FBA3	true	Sedoheptulose1, 7-bisphosphateD-glyceraldehyde-3--phosphate-lyase	[c] : s17bp <=> dhap + e4p
Fructose and Mannose Metabolism	4.1.2.13	R_FBA2	true	D-Fructose1-phosphate-D-glyceraldehyde-3--phosphate-lyase	[c] : f1p <=> dhap + glyald
Glycolysis/Gluconeogenesis	4.1.2.13	R_FBA	true	fructose-bisphosphate-aldolase	[c] : fdp <=> dhap + g3p
Glycolysis/Gluconeogenesis	4.2.1.11	R_ENO	true	enolase	[c] : 2pg <=> h2o + pep
Glycoprotein Metabolism	2.4.1.131	R_G12MT2g	false	Glycolipid1, 2-alpha-D-mannosyltransferase, Golgiapparatus	[g] : gdpmann + m3macchitppdol → gdp + h + m4macchitppdol
Glycoprotein Metabolism	2.4.1.131	R_G12MT1g	false	Glycolipid1, 2-alpha-D-mannosyltransferase, Golgiapparatus	[g] : gdpmann + m2macchitppdol → gdp + h + m3macchitppdol
Glycoprotein Metabolism	2.4.1.109	R_DOLPMMer	false	Dolichyl-phosphate--mannose–proteinmannosyltransferase, endoplasmicreticular	[r] : dolmanp → dolp + h + mannan
Histidine Metabolism	3.5.3.19	R_UGLYCHr	true	Ureidoglycolate-hydrolase	[c] : 2.0 h + h2o + urdglyc <=> 1.0 co2 + 1.0 glx + 2.0 nh4
Pentose Phosphate Pathway	2.4.2.17	R_PRPPS	true	phosphoribosylpyrophosphatesynthetase	[c] : atp + r5p <=> amp + h + prpp
Histidine Metabolism	5.3.1.16	R_PRMICIi	false	_1- (5-phosphoribosyl)-5-[(5-phosphoribosylamino)methylideneamino)imidazole-4-carboxamideisomerase (irreversible)	[c] : prfp → prlp
Histidine Metabolism	3.5.4.19	R_PRATPP	false	phosphoribosyl-ATPpyrophosphatase	[c] : h2o + prbatp → h + ppi + prbamp
Histidine Metabolism	3.5.4.19	R_PRAMPC	false	phosphoribosyl-AMPcyclo-hydrolase	[c] : h2o + prbamp → prfp
Histidine Metabolism	4.2.1.19	R_IGPDH	false	imidazoleglycerol--phosphatedehydratase	[c] : eig3p → h2o + imacp
Histidine Metabolism	2.4.2.-	R_IG3PS	false	Imidazole-glycerol-3--phosphatesynthase	[c] : gln_l + prlp → aicar + eig3p + glu_l + h
Histidine Metabolism	2.6.1.9	R_HSTPT	false	histidinol-phosphate-transaminase	[c] : glu_l + imacp → akg + hisp
Histidine Metabolism	6.1.1.21	R_HISTRS	false	Histidyl-tRNAsynthetase	[c] : atp + his_l + trnahis → amp + histrna + ppi
Histidine Metabolism	6.1.1.21	R_HISTRSm	false	histidyl-tRNAsynthetase, mitochondrial	[m] : atp + his_l + trnahis → amp + histrna + ppi
Histidine Metabolism	3.1.3.15	R_HISTP	false	histidinol-phosphatase	[c] : h2o + hisp → histd + pi
Histidine Metabolism	1.1.1.23	R_HISTD	false	histidinoldehydrogenase	[c] : h2o + histd + 2.0 nad → 3.0 h + 1.0 his_l + 2.0 nadh
Histidine Metabolism	2.4.2.17	R_ATPPRT	false	ATPphosphoribosyl-transferase	[c] : atp + prpp → ppi + prbatp

Histidine Metabolism	2.1.1.-	R_ARMT	false	hnRNPargininеN--methyltransferase	[c] : amet + his_l → npmehis + ahcys + h
Histidine Metabolism	3.5.2.5	R_ALLTNr	true	allantoinase, reversible-reaction	[c] : alltn + h2o <=> alltt + h
Histidine Metabolism	3.5.3.4	R_ALLTAHr	true	Allantoateamidino-hydrolase, reversible	[c] : alltt + h2o <=> urdglyc + urea
Fatty Acid Metabolism	1.2.1.2	R_FDH	false	formatedehydrogenase	[c] : for + nad → co2 + nadh
Methionine Metabolism	4.2.99.9	R_SHSL4r	true	O-succinylhomoserinelyase (elimination), -reversible	[c] : h2o + suchms <=> 2obut + h + nh4 + succ
Methionine Metabolism	2.5.1.48	R_SHSL1	false	O-succinylhomoserinelyase (L-cysteine)	[c] : cys_l + suchms → cyst_l + h + succ
Methionine Metabolism	2.1.1.14	R_MHPGLUT	false	_5-methyl-tetrahydropteroyltriglutamate-homocysteineS-methyl-transferase	[c] : hcys_l + mhpglu → hpglu + met_l
Methionine Metabolism	6.1.1.10	R_METTRSm	false	methionyl-tRNAsynthetase, mitochondrial	[m] : atp + met_l + trnamet → amp + mettrna + ppi
Methionine Metabolism	6.1.1.10	R_METTRS	false	Methionyl-tRNAsynthetase	[c] : atp + met_l + trnamet → amp + mettrna + ppi
Methionine Metabolism	2.1.1.13	R_METS	false	methioninesynthase	[c] : 5mthf + hcys_l → h + met_l + thf
Methionine Metabolism	4.2.99.9	R_METB1	false	metb1	[c] : achms + cys_l → ac + cyst_l + h
Methionine Metabolism	2.5.1.6	R_METAT	false	methionineadenosyl-transferase	[c] : atp + h2o + met_l → amet + pi + ppi
Methionine Metabolism	2.3.1.31	R_HSERTA	true	homoserineO-trans-acetylase	[c] : accoa + hom_l <=> achms + coa
Methionine Metabolism	2.1.1.98	R_DIPS	false	diphthinesynthase	[c] : amet + caphis → ahcys + cmaphis + h
Methionine Metabolism	4.4.1.8	R_CYSTLp	false	cystathioneb-lyase, per-oxisomal	[x] : cyst_l + h2o → hcys_l + nh4 + pyr
Methionine Metabolism	4.4.1.8	R_CYSTL	false	cystathionineb-lyase	[c] : cyst_l + h2o → hcys_l + nh4 + pyr
Cysteine Metabolism	4.4.1.1	R_CYSTGL	false	cystathionineg-lyase	[c] : cyst_l + h2o → 2obut + cys_l + nh4
Methionine Metabolism	4.2.99.10	R_AHSERL2	false	O-acetylhomoserine (thiol)-lyase	[c] : achms + h2s → ac + h + hcys_l
Methionine Metabolism	4.2.99.10	R_AHSERL	false	O-acetylhomoserine (thiol)-lyase	[c] : achms + ch4s → ac + h + met_l
Methionine Metabolism	3.3.1.1	R_AHCi	false	adenosylhomocysteinase	[c] : ahcys + h2o → adn + hcys_l
NAD Metabolism	2.7.7.18	R_NNATm	false	nicotinate-nucleotide-adenylyltransferase, mitochondrial	[m] : atp + h + nicrnt → dnad + ppi
NAD Metabolism	2.7.7.18	R_NNAT	false	nicotinate-nucleotide-adenylyltransferase	[c] : atp + h + nicrnt → dnad + ppi
NAD Biosyn-thesis	3.5.1.19	R_NNAMrm	true	nicotinamidase, -reversible, mitochon-drial	[m] : h2o + ncam <=> nac + nh4
NAD Biosyn-thesis	3.5.1.19	R_NNAMr	true	nicotinamidase, -reversible	[c] : h2o + ncam <=> nac + nh4
NAD Metabolism	2.7.7.1	R_NMNATm	false	nicotinamide--nucleotideadenylyl-transferase, mitochon-drial	[m] : atp + h + nmn → nad + ppi
NAD Biosyn-thesis	6.3.1.5	R_NADS1	false	NADsynthase (nh3)	[c] : atp + dnad + nh4 → amp + h + nad + ppi
NAD Biosyn-thesis	6.3.1.5	R_NADS1m	false	NADsynthase (nh3), mi-tochondrial	[m] : atp + dnad + nh4 → amp + h + nad + ppi
NAD Biosyn-thesis	2.7.1.23	R_NADKm	false	NADkinase, mitochon-drial	[m] : atp + nad → adp + h + nadp
NAD Metabolism	2.7.1.23	R_NADK	false	NADkinase	[c] : atp + nad → adp + h + nadp
Nitrogen Metabolism	6.3.4.6	R_UREASE	true	ureacarboxylase	[c] : atp + hco3 + urea <=> adp + allphn + h + pi
Nitrogen Metabolism	3.5.5.1	R_NTRLASE	false	nitrilase	[c] : 2.0 h2o + ind3acnl → ind3ac + nh4
Nitrogen Metabolism	3.5.1.54	R_ALPHNH	false	allophanate-hydrolase	[c] : allphn + 3.0 h + h2o → 2.0 co2 + 2.0 nh4
Nucleotide Salvage Pathway	2.4.2.22	R_XPPT	false	xanthinephospho-ribosyltransferase	[c] : prpp + xan → ppi + xmp
Nucleotide Salvage Pathway	2.7.4.-	R_URIDK2rn	true	uridylatekinase (dUMP), nuclear	[n] : atp + dump <=> adp + dudp
Nucleotide Salvage Pathway	2.7.4.-	R_URIDK2r	true	uridylatekinase (dUMP)	[c] : atp + dump <=> adp + dudp
Nucleotides	2.7.4.14	R_UMPK	true	UMPkinase	[c] : atp + ump <=> adp + udp
Nucleotides	2.7.4.14	R_UMPKn	true	UMPkinase, nuclear	[n] : atp + ump <=> adp + udp
Nucleotides	2.7.4.6	R_NDPK9	true	nucleoside-diphosphate-kinase (ATP:IDP)	[c] : atp + idp <=> adp + itp

Nucleotides	2.7.4.6	R_NDPK8	true	nucleoside-diphosphate-kinase (ATP:dADP)	[c] : atp + dadp <=> adp + datp
Nucleotides	2.7.4.6	R_NDPK7	true	nucleoside-diphosphate-kinase (ATP:dCDP)	[c] : atp + dcdp <=> adp + dctp
Nucleotides	2.7.4.6	R_NDPK6	true	nucleoside-diphosphate-kinase (ATP:dUDP)	[c] : atp + dudp <=> adp + dutp
Nucleotides	2.7.4.6	R_NDPK5	true	nucleoside-diphosphate-kinase (ATP:dGDP)	[c] : atp + dgdp <=> adp + dgtp
Nucleotides	2.7.4.6	R_NDPK4	true	nucleoside-diphosphate-kinase (ATP:dTDP)	[c] : atp + dtdp <=> adp + dttp
Nucleotides	2.7.4.6	R_NDPK3	true	nucleoside-diphosphate-kinase (ATP:CDP)	[c] : atp + cdp <=> adp + ctp
Nucleotides	2.7.4.6	R_NDPK2	true	nucleoside-diphosphate-kinase (ATP:UDP)	[c] : atp + udp <=> adp + utp
Nucleotides	2.7.4.6	R_NDPK1	true	nucleoside-diphosphate-kinase (ATP:GDP)	[c] : atp + gdp <=> adp + gtp
Salvage Pathway	2.4.2.8	R_HXPRT	false	hypoxanthinephospho-ribosyltransferase (Hypoxanthine)	[c] : hxan + prpp → imp + ppi
Nucleotide Salvage Pathway	.	R_GNNUC	false	gnnuc	[c] : gsn + h2o → gua + rib_d
Pyrimidine Catabolism	3.5.4.14	R_DCYTD	false	deoxycytidinedeaminase	[c] : dcyt + h + h2o → duri + nh4
Nucleotide Salvage Pathway	3.5.4.12	R_DCTPD	false	dCTPdeaminase	[c] : dctp + h + h2o → dutp + nh4
Nucleotides	2.7.4.14	R_CYTK2	true	cytidylatekinase (dCMP)	[c] : atp + dcmp <=> adp + dcdp
Nucleotides	2.7.4.14	R_CYTK1	true	cytidylatekinase (CMP)	[c] : atp + cmp <=> adp + cdp
Nucleotide Salvage Pathway	2.7.1.48	R_CYTDK2	false	cytidinekinase (GTP)	[c] : cytd + gtp → cmp + gdp + h
Nucleotide Salvage Pathway	3.2.2.4	R_AMPN	false	AMPnucleosidase	[c] : amp + h2o → ade + r5p
Nucleotide Salvage Pathway	3.2.2.8	R_ADNUC	false	adenosine-hydrolase	[c] : adn + h2o → ade + rib_d
Nucleotide Salvage Pathway	2.7.4.3	R_ADK4	true	adentylatekinase (ITP)	[c] : amp + itp <=> adp + idp
Nucleotide Salvage Pathway	2.7.4.3	R_ADK4m	true	adentylatekinase (ITP), mitochondrial	[m] : amp + itp <=> adp + idp
Nucleotides	.	R_ADK3	true	adentylatekinase (GTP)	[c] : amp + gtp <=> adp + gdp
Nucleotides	.	R_ADK3m	true	adentylatekinase (GTP)	[m] : amp + gtp <=> adp + gdp
Nucleotides	2.7.4.10	R_ADK1m	true	adenylatekinase, mito-chondrial	[m] : amp + atp <=> 2.0 adp
Nucleotides	2.7.4.3	R_ADK1	true	adenylatekinase	[c] : amp + atp <=> 2.0 adp
Salvage Pathway	2.4.2.7	R_GUAPRT	false	guaninephosphoribosyl-transferase	[c] : gua + prpp → gmp + ppi
Other Amino Acid Metabolism	2.3.1.88	R_PEPAT	false	Peptidealpha-N-acetyl-transferase	[c] : accoa + pepd → apep + coa + h
Other Amino Acid Metabolism	3.5.5.1	R_NTRLASE3	false	nitrilase	[c] : acybut + 2.0 h2o → glu_l + nh4
Other Amino Acid Metabolism	3.5.5.1	R_NTRLASE2	false	nitrilase	[c] : aprop + 2.0 h2o → ala_l + nh4
Arginine and Proline Metabolism	1.2.1.3	R_NABTNO	false	N4-Acetylaminobutanal:NAD+oxido-reductase	[c] : h2o + n4abutn + nad → 4aabutn + h + nadh
Other Amino Acid Metabolism	1.2.1.21	R_LCADm	true	lactaldehydede-hydrogenase, mito-chondrial	[m] : h2o + lald_l + nad <=> h + lac_l + nadh
Other Amino Acid Metabolism	2.3.2.2	R_GTMLT	false	g-glutamyltransferase	[c] : ala_l + gthrd → cgly + gluala
Glutathione Metabolism	6.3.2.3	R_GTHS	false	glutathionesynthetase	[c] : atp + glucys + gly → adp + gthrd + h + pi
Glutathione Metabolism	1.11.1.9	R_GTHP	true	glutathioneperidoxase	[c] : 2.0 gthrd + h2o2 <=> 1.0 gthox + 2.0 h2o
Glutamate metabolism	1.6.4.2	R_GTHO	false	glutathioneoxido-reductase	[c] : gthox + h + nadph → gthrd + nadp
Glutathione Metabolism	6.3.2.2	R_GLUCYS	false	gamma-glutamylcysteinesynthetase	[c] : atp + cys_l + glu_l → adp + glucys + h + pi
Other Amino Acid Metabolism	2.3.1.97	R_GLPT	false	glycylpeptideN-tetradecanoyl-transferase	[c] : glp + tdcoa → coa + tglp
Other Amino Acid Metabolism	1.2.1.19	R_ABUTDm	false	Aminobutyraldehydede-hydrogenase, mitochon-drial	[m] : 4abutn + h2o + nad → 4abut + h + nadh
Oxidative Phosphory-lation	1.3.5.1	R_SUCD3_DASH_u6m	true	succinatedehydrogenase (ubiquinone-6), mito-chondrial	[m] : fadh2 + q6 <=> fad + q6h2
Citric Acid Cycle	1.3.5.1	R_SUCD1m	true	succinatedehydrogenase	[m] : fad + succ <=> fadh2 + fum

Oxidative Phosphorylation	3.6.1.1	R_PPAm	false	inorganicdiphosphatase	[m] : h2o + ppi → 1.0 h + 2.0 pi
Oxidative Phosphorylation	3.6.1.1	R_PPA	false	inorganicdiphosphatase	[c] : h2o + ppi → 1.0 h + 2.0 pi
Oxidative Phosphorylation	1.6.5.3	R_NADH2_DASH_u6m	false	NADHdehydrogenase, mitochondrial	[m] : h + nadh + q6 → nad + q6h2
Oxidative Phosphorylation	1.6.99.3	R_NADH2_DASH_u6cm	false	NADHdehydrogenase, cytosolic/mitochondrial	h[c] + nadh[c] + q6[m] → nad[c] + q6h2[m]
Oxidative Phosphorylation	1.1.1.37	R_MDHp	true	malatedehydrogenase, peroxisomal	[x] : mal_l + nad <=> h + nadh + oaa
Citric Acid Cycle	1.1.1.37	R_MDHm	true	malatedehydrogenase, mitochondrial	[m] : mal_l + nad <=> h + nadh + oaa
Citric Acid Cycle	1.1.1.37	R_MDH	true	malatedehydrogenase	[c] : mal_l + nad <=> h + nadh + oaa
Citric Acid Cycle	4.2.1.2	R_FUM	true	fumarase	[c] : fum + h2o <=> mal_l
Citric Acid Cycle	4.2.1.2	R_FUMm	true	fumarase, mitochondrial	[m] : fum + h2o <=> mal_l
Oxidative Phosphorylation	1.10.2.2	R_CYOR_u6m	false	ubiquinol-6cytochromecreductase	2.0 ficytc[m] + 1.5 h[m] + q6h2[m] → focytc[m] + h[c] + q6[m]
Oxidative Phosphorylation	1.9.3.1	R_CYOOm	false	cytochromecoxidase, mitochondrial	4.0 focytc[m] + 6.0 h[m] + o2[m] → 4.0 ficytc[m] + 6.0 h[c] + 2.0 h2o[m]
Oxidative Phosphorylation	3.6.3.14	R_ATPS3v	false	ATPsynthase, vacuole	adp[v] + 3.0 h[c] + pi[v] → atp[v] + h[v] + h2o[v]
Oxidative Phosphorylation	3.6.3.14	R_ATPS3m	false	ATPsynthase, mitochondrial	adp[m] + 3.0 h[c] + pi[m] → atp[m] + h[m] + h2o[m]
Pantothenate and CoA Biosynthesis	6.3.2.1	R_PANTS	false	pantothenatesynthase	[c] : ala_b + atp + pant_r → amp + h + pnto_r + ppi
Pantothenate and CoA Biosynthesis	1.1.1.169	R_DPRm	false	_2-dehydropantoate2-reductase, mitochondrial	[m] : 2dhp + h + nadph → nadp + pant_r
Pantothenate and CoA Biosynthesis	2.8.1.6	R_BTSr	true	biotinsynthase	[c] : dtbt + s <=> 1.0 btn + 2.0 h
Pantothenate and CoA Biosynthesis	2.6.1.62	R_AMAOTr	true	adenosylmethionine-8-amino-7-oxononanoatetransaminase	[c] : 8aonn + amet <=> amob + dann
Pentose Phosphate Pathway	2.2.1.1	R_TKT2	true	transketolase	[c] : e4p + xu5p_d <=> f6p + g3p
Pentose Phosphate Pathway	2.2.1.1	R_TKT1	true	transketolase	[c] : r5p + xu5p_d <=> g3p + s7p
Pentose Phosphate Pathway	2.2.1.2	R_TALA	true	transaldolase	[c] : g3p + s7p <=> e4p + f6p
Pentose Phosphate Pathway	5.1.3.1	R_RPE	true	ribulose5-phosphate3-epimerase	[c] : ru5p_d <=> xu5p_d
Pentose Phosphate Pathway	2.7.1.15	R_RBK	false	ribokinase	[c] : atp + rib_d → adp + h + r5p
Pentose Phosphate Pathway	5.4.2.2	R_PPM	true	phosphopentomutase	[c] : r1p <=> r5p
Glycolysis/Gluconeogenesis	5.4.2.2	R_PGMT	true	phosphoglucomutase	[c] : g1p <=> g6p
Pentose Phosphate Pathway	3.1.1.31	R_PGL	false	_6-phosphogluconolactonase	[c] : 6pgl + h2o → 6pgc + h
Pentose Phosphate Pathway	1.1.1.43	R_GND	false	phosphogluconatedehydrogenase	[c] : 6pgc + nadp → co2 + nadph + ru5p_d
Pentose Phosphate Pathway	1.1.1.49	R_G6PDH2	false	glucose6-phosphatedehydrogenase	[c] : g6p + nadp → 6pgl + h + nadph
Pentose Phosphate Pathway	2.7.1.15	R_DRBK	false	Deoxyribokinase	[c] : atp + drib → 2dr5p + adp + h
Phospholipid Biosynthesis	2.7.8.8	R_PSERSm_SC	true	phosphatidylserinesynthase, yeast-specific, mitochondrial	[m] : 0.01 cdpdag_sc + ser_l <=> 1.0 cmp + 1.0 h + 0.01 ps_sc
Phospholipid Biosynthesis	2.7.8.8	R_PSERS_SC	true	phosphatidylserinesynthase, yeast-specific	[c] : 0.01 cdpdag_sc + ser_l <=> 1.0 cmp + 1.0 h + 0.01 ps_sc
Phospholipid Biosynthesis	4.1.1.65	R_PSERDv_SC	false	phosphatidylserinedecarboxylase, yeast-specific, vacuolar	[v] : h + 0.01 ps_sc → 1.0 co2 + 0.01 pe_sc

Phospholipid Biosynthesis	4.1.1.65	R_PSERDg_SC	false	phosphatidylserinedecarboxylase, yeast-specific, Golgi	[g] : h + 0.01 ps_sc → 1.0 co2 + 0.01 pe_sc
Phospholipid Biosynthesis	4.1.1.65	R_PSERDm_SC	false	phosphatidylserinedecarboxylase, yeast-specific, mitochondrial	[m] : h + 0.01 ps_sc → 1.0 co2 + 0.01 pe_sc
Phospholipid Biosynthesis	2.1.1.16	R_PMETM_SC	false	Phosphatidyl-N-methyl-ethanolamineN-methyl-transferase, yeast-specific	[c] : amet + 0.01 ptd2meeta_sc → 1.0 ahcys + 1.0 h + 0.01 pc_sc
Phospholipid Biosynthesis	2.7.1.67	R_PIN4K_SC	false	phosphatidylinositol4-kinase, yeast-specific	[c] : atp + 0.01 ptd1ino_sc → 1.0 adp + 1.0 h + 0.01 ptd4ino_sc
Phospholipid Biosynthesis	2.7.1.67	R_PIN4Kn_SC	false	phosphatidylinositol4-kinase, nuclear, yeast-specifc	[n] : atp + 0.01 ptd1ino_sc → 1.0 adp + 1.0 h + 0.01 ptd4ino_sc
Phospholipid Biosynthesis	2.7.1.137	R_PIN3K_SC	false	_1-phosphatidylinositol3-kinase, yeast-specific	[c] : atp + 0.01 ptd1ino_sc → 1.0 adp + 1.0 h + 0.01 ptd3ino_sc
Phospholipid Biosynthesis	3.1.4.11	R_PI45BPP_SC	false	_1-phosphatidylinositol-4, 5-bisphosphate-phosphodiesterase, yeast-specific	[c] : h2o + 0.01 ptd145bp_sc → 12dgr_sc + h + mi145p
Phospholipid Biosynthesis	2.1.1.17	R_PETOHM_SC	false	phosphatidylethanolamineN-methyltransferase, yeast-specific	[c] : amet + 0.01 pe_sc → 1.0 ahcys + 1.0 h + 0.01 ptdmeeta_sc
Inositol Phosphate Metabolism	5.5.1.4	R_MI1PS	false	myo-Inositol-1--phosphatesynthase	[c] : g6p → mi1p_d
Phospholipid Biosynthesis	2.1.1.16	R_MFAPS_SC	false	methylene-fatty-acyl--phospholipidsynthase, yeast-specific	[c] : amet + 0.01 ptdmeeta_sc → 1.0 ahcys + 1.0 h + 0.01 ptd2meeta_sc
Phospholipid Biosynthesis	.	R_LPP_SC	false	lipidphosphate-phosphatase, yeast-specific	[c] : 0.01 dagpy_sc + h2o → h + pa_sc + pi
Phospholipid Biosynthesis	2.7.8.1	R_ETHAPT_SC	true	Ethanolaminephosphotransferase, yeast-specific	[c] : 0.01 12dgr_sc + cdpea <=> 1.0 cmp + 1.0 h + 0.01 pe_sc
Glycerophospholipid Metabolism	2.7.1.32	R_ETHAK	false	Ethanolaminekinase	[c] : atp + etha → adp + ethamp + h
Phospholipid Biosynthesis	3.1.3.4	R_DAGPYP_SC	false	diacylglycerolpyrophosphatephosphatase, yeast-specific	[c] : h2o + 0.01 pa_sc → 12dgr_sc + pi
Phospholipid Biosynthesis	2.7.8.1	R_DAGCPT_SC	false	diacylglycerolcholinephosphotransferase, yeast-specific	[c] : 0.01 12dgr_sc + cdpchol → 1.0 cmp + 1.0 h + 0.01 pc_sc
Phospholipid Biosynthesis	2.7.8.5	R_CLPNSm_SC	false	cardiolipinsynthase, yeast-specific, mitochondrial	[m] : 0.01 cdpdag_sc + 0.01 pg_sc → clpn_sc + cmp + h
Glycerophospholipid Metabolism	2.7.1.32	R_CHOLK	false	Cholinekinase	[c] : atp + chol → adp + cholp + h
Phospholipid Biosynthesis	2.7.8.8	R_CDPDGPm_SC	true	CDP-diacylglycerol–serineO-phosphatidyltransferae, yeast-specific, mitochondrial	[m] : 0.01 cdpdag_sc + glyc3p <=> 1.0 cmp + 1.0 h + 0.01 pgp_sc
Phospholipid Biosynthesis	2.3.1.51	R_AGAT_SC	false	_1-Acyl-glycerol-3-phosphateacyl-transferase, yeast-specific	[c] : 0.01 1ag3p_sc + 0.02 dcacoa + 0.06 ddcacoa + 0.17 hdcoa + 0.09 ocdycacoa + 0.24 odecoa + 0.27 pmtcoa + 0.05 stcoa + 0.1 tdcoa → 1.0 coa + 0.01 pa_sc
Heme Biosynthesis	4.1.1.37	R_UPPDC1	false	uroporphyrinogendecarboxylase (uroporphyrinogenIII)	[c] : 4.0 h + uppg3 → co2 + cpppg3
Heme Biosynthesis	4.2.1.24	R_PPBNGS	false	porphobilinogensynthase	[c] : 2.0 5aop → h + h2o + ppbng
Heme Biosynthesis	4.3.1.8	R_HMBS	false	hydroxymethyl-bilanesynthase	[c] : h2o + 4.0 ppbng → 1.0 hmbil + 4.0 nh4
Heme Biosynthesis	1.3.3.3	R_CPPPGO	false	coproporphyrinogenoxidase (O2required)	[c] : cpppg3 + 2.0 h + o2 → co2 + h2o + pppg9
Glycine, Serine, and Threonine Metabolism	2.3.1.29	R_ALASm	false	_5-aminolevulinatesynthase	[m] : gly + h + succoa → 5aop + co2 + coa
Purine and Pyrimidine Biosynthesis	4.2.1.70	R_YUMPS	true	yUMPsynthetase	[c] : r5p + ura <=> h2o + psd5p
Purine and Pyrimidine Biosynthesis	2.7.1.48	R_URIK2	false	uridinekinase (GTP:Uridine)	[c] : gtp + uri → gdp + h + ump
Pyrimidine Biosynthesis	2.7.1.48	R_URIK1	false	uridinekinase (ATP:Uridine)	[c] : atp + uri → adp + h + ump

Purine and Pyrimidine Biosynthesis	2.4.2.9	R_UPPRT	false	uracilphosphoribosyltransferase	[c] : prpp + ura $\rightarrow$ ppi + ump
Nucleotides	1.8.1.9	R_TRDR	false	thioredoxinreductase (NADPH)	[c] : h + nadph + trdox $\rightarrow$ nadp + trdrd
Nucleotides	2.1.1.45	R_TMDS	false	thymidylatesynthase	[c] : dump + mlthf $\rightarrow$ dhf + dtmp
Purine and Pyrimidine Biosynthesis	3.1.4.17	R_PDE5	false	_3', 5'-cyclic-nucleotide-phosphodiesterase	[c] : 35ccmp + h2o $\rightarrow$ cmp + h
Nucleotides	3.1.4.17	R_PDE4	false	_3', 5'-cyclic-nucleotide-phosphodiesterase	[c] : 35cgmp + h2o $\rightarrow$ gmp + h
Purine and Pyrimidine Biosynthesis	3.1.4.17	R_PDE3	false	_3', 5'-cyclic-nucleotide-phosphodiesterase	[c] : 35cimp + h2o $\rightarrow$ h + imp
Purine and Pyrimidine Biosynthesis	3.1.4.17	R_PDE2	false	_3', 5'-cyclic-nucleotide-phosphodiesterase	[c] : 35cdamp + h2o $\rightarrow$ damp + h
Nucleotides	3.1.4.17	R_PDE1	false	_3', 5'-cyclic-nucleotide-phosphodiesterase	[c] : camp + h2o $\rightarrow$ amp + h
Pyrimidine Biosynthesis	2.4.2.10	R_ORPT	true	orotatephosphoribosyltransferase	[c] : orot5p + ppi <=> orot + prpp
Nucleotides	1.1.1.205	R_IMPD	false	IMPdehydrogenase	[c] : h2o + imp + nad $\rightarrow$ h + nadh + xmp
IMP Biosynthesis	3.5.4.10	R_IMPC	true	IMPcyclo-hydrolase	[c] : h2o + imp <=> fprica
Nucleotides	6.3.5.2	R_GMPS2	false	GMPsynthase	[c] : atp + gln_l + h2o + xmp $\rightarrow$ amp + glu_l + gmp + h + ppi
IMP Biosynthesis	2.4.2.14	R_GLUPRT	false	glutaminephosphoribosyldiphosphate-amidotransferase	[c] : gln_l + h2o + prpp $\rightarrow$ glu_l + ppi + pram
Purine and Pyrimidine Biosynthesis	2.7.4.8	R_GK2	true	guanylatekinase (GMP:dATP)	[c] : datp + gmp <=> dadp + gdp
Nucleotides	2.7.4.8	R_GK1	true	guanylatekinase (GMP:ATP)	[c] : atp + gmp <=> adp + gdp
Purine and Pyrimidine Biosynthesis	2.1.2.2	R_GARFTi	false	phosphoribosylglycinamideformyltransferase, irreversible	[c] : 10fthf + gar $\rightarrow$ fgam + h + thf
Purine and Pyrimidine Biosynthesis	3.6.1.23	R_DUTPDP	false	dUTPdiphosphatase	[c] : dutp + h2o $\rightarrow$ dump + h + ppi
Purine and Pyrimidine Biosynthesis	3.5.2.3	R_DHORTSn	true	dihydroorotase, nuclear	[n] : dhor_s + h2o <=> cbasp + h
Purine and Pyrimidine Biosynthesis	1.3.3.1	R_DHORD4	true	dihydooroticacid (ubiquinone-6)	dhor_s[c] + q6[m] <=> orot[c] + q6h2[m]
Purine and Pyrimidine Biosynthesis	1.3.3.1	R_DHORD	true	dihydooroticaciddehydrogenase	[c] : dhor_s + o2 <=> h2o2 + orot
Nucleotides	2.7.4.8	R_DGK1	true	deoxyguanylatekinase (dGMP:ATP)	[c] : atp + dgmp <=> adp + dgdp
Pyrimidine Catabolism	3.5.4.12	R_DCMPDA	true	dCMPdeaminase	[c] : dcmp + h + h2o <=> dump + nh4
Pyrimidine Catabolism	3.5.4.5	R_CYTD	false	cytidinedeaminase	[c] : cytd + h + h2o $\rightarrow$ nh4 + uri
Nucleotides	6.3.4.2	R_CTPS1	false	CTPsynthase (NH3)	[c] : atp + nh4 + utp $\rightarrow$ adp + ctp + h + pi
Pyrimidine Biosynthesis	6.3.4.2	R_CTPS2	false	CTPsynthase (glutamine)	[c] : atp + gln_l + h2o + utp $\rightarrow$ adp + ctp + glu_l + h + pi
Pyrimidine Catabolism	3.5.4.1	R_CSND	false	Cytosinedeaminase	[c] : csn + h + h2o $\rightarrow$ nh4 + ura
Purine and Pyrimidine Biosynthesis	2.1.3.2	R_ASPCTn	false	aspartatecarbamoyltransferase, nuclear	[n] : asp_l + cbp $\rightarrow$ cbasp + h + pi
Nucleotides	3.5.4.6	R_AMPDA	false	Adenosinemonophosphatedeaminase	[c] : amp + h + h2o $\rightarrow$ imp + nh4
IMP Biosynthesis	4.1.1.21	R_AIRCr	true	phosphoribosylaminoimidazolecarboxylase	[c] : air + co2 <=> 5aizc + h
IMP Biosynthesis	2.1.2.3	R_AICART	true	phosphoribosylaminoimidazolecarboxamideformyltransferase	[c] : 10fthf + aicar <=> fprica + thf
Nucleotides	6.3.4.4	R_ADSS	false	adenylosuccinatesynthase	[c] : asp_l + gtp + imp $\rightarrow$ dcamp + gdp + h + pi
Purine and Pyrimidine Biosynthesis	4.3.2.2	R_ADSL2r	true	adenylosuccinatelyase	[c] : 25aics <=> aicar + fum

Purine and Pyrimidine Biosynthesis	4.3.2.2	R_ADSL1r	true	adenylsuccinatelyase	[c] : dcamp <=> amp + fum
Nucleotides	4.6.1.1	R_ADNCYC	false	adenylatecyclase	[c] : atp → camp + ppi
Pyruvate Metabolism	4.1.1.1	R_PYRDC	false	pyruvatedecarboxylase	[c] : h + pyr → acald + co2
Pyruvate Metabolism	4.1.3.21	R_HCITSn	false	homocitratesynthase	[n] : accoa + akg + h2o → coa + h + hcit
Pyruvate Metabolism	4.1.3.21	R_HCITSm	false	homocitratesynthase	[m] : accoa + akg + h2o → coa + h + hcit
Tyrosine metabolism	1.1.1.1	R_FALDH	true	formaldehydedehydrogenase	[c] : fald + gthrd + nad <=> sfglutth + h + nadh
Pyruvate Metabolism	1.1.1.1	R_ALCD2x	true	alcoholdehydrogenase (ethanol)	[c] : etoh + nad <=> acald + h + nadh
Glycolysis/Gluconeogenesis	6.2.1.1	R_ACS	false	acetyl-CoAsynthetase	[c] : ac + atp + coa → accoa + amp + ppi
Glycolysis/Gluconeogenesis	6.2.1.1	R_ACSm	false	acetyl-CoAsynthetase	[m] : ac + atp + coa → accoa + amp + ppi
Pyruvate Metabolism	6.2.1.1	R_ACSp	false	acetyl-CoAsynthetase	[x] : ac + atp + coa → accoa + amp + ppi
Quinone Biosynthesis	1.14.13.-	R_OMMBLHX6m	false	_2-Octaprenyl-3--methyl-6-methoxy-1, 4-benzoquinolhydroxylase, mitochodnrial	[m] : 2ommbl_5 + 0.5 o2 → 2omhmbl_5
Quinone Biosynthesis	2.5.1.-	R_HBZOPT6m	false	Hydroxybenzoateoctaprenyltransferase, mitochondrial	[m] : 4hbz + octdp_5 → 3ophb_5 + ppi
Quinone Biosynthesis	2.5.1.-	R_HBZOPT6	false	Hydroxybenzoateoctaprenyltransferase	[c] : 4hbz + octdp_5 → 3ophb_5 + ppi
Quinone Biosynthesis	4.1.3.-	R_CHRPL	false	Chorismatepyruvatelyase	[c] : chor → 4hbz + pyr
Riboflavin Metabolism	1.1.1.193	R_DHPPDA	false	diaminohydroxy-phospho-ribosylaminopyrimidinedeaminase	[c] : 25dhpp + h + h2o → 5apru + nh4
Riboflavin Metabolism	1.1.1.193	R_APRAUR	false	_5-amino-6- (5-phosphoribosylamino)uracilreductase	[c] : 5apru + h + nadph → 5aprbu + nadp
Riboflavin Metabolism	3.1.3.2	R_ACP1e	false	acidphosphatase, extracellular (secreted)	[e] : fmn + h2o → pi + ribflv
Starch and Sucrose Metabolism	3.2.1.58	R_13BGHe	false	Exo-1, 3-beta-glucanglucohydrase	[e] : 13bdglcn + h2o → glc_d
Thiamine Metabolism	2.5.1.3	R_TMPPP	false	thiamine-phosphatediphosphorylase	[c] : 2mahmp + 4mpetz + h → ppi + thmmp
Thiamine Metabolism	3.1.3.2	R_THMPe	false	thiaminphosphatase, extracellular	[e] : h2o + thmmp → pi + thm
Thiamine Metabolism	3.1.3.2	R_THMDPe	false	thiamindiphosphatase, extracellular	[e] : 2.0 h2o + thmpp → h + pi + thm
Thiamine Metabolism	2.7.4.7	R_PMPK	false	phosphomethyl-pyrimidinekinase	[c] : 4ampm + atp → 2mahmp + adp
Thiamine Metabolism	2.7.1.49	R_HMPK1	false	hydroxymethyl-pyrimidinekinase (ATP)	[c] : 4ahmmp + atp → 4ampm + adp + h
Thiamine Metabolism	2.5.1.3	R_HETZK	false	hydroxyethylthiazolekinase	[c] : 4mhetz + atp → 4mpetz + adp + h
Threonine and Lysine Metabolism	6.1.1.3	R_THRTRS	false	Threonyl-tRNAsynthetase	[c] : atp + thr_l + trnathr → amp + ppi + thrtrna
Threonine and Lysine Metabolism	6.1.1.3	R_THRTRSm	false	threonyl-tRNAsynthetase, mitochondrial	[m] : atp + thr_l + trnathr → amp + ppi + thrtrna
Glycine, Serine, and Threonine Metabolism	4.2.3.1	R_THRS	false	threoninesynthase	[c] : h2o + phom → pi + thr_l
Glycine, Serine, and Threonine Metabolism	4.2.1.16	R_THRD_L	false	L-threoninedeaminase	[c] : thr_l → 2obut + nh4
Threonine and Lysine Metabolism	4.2.1.16	R_THRD_Lm	false	L-threoninedeaminase, mitochondrial	[m] : thr_l → 2obut + nh4
Threonine and Lysine Metabolism	4.1.2.5	R_THRA	false	Threoninealdolase	[c] : acald + gly → thr_l
Threonine and Lysine Metabolism	1.5.1.7	R_SACCD2	true	saccharopinedehydrogenase (NAD, L-lysineforming)	[c] : h2o + nad + saccrp_l <=> akg + h + lys_l + nadh
Threonine and Lysine Metabolism	1.5.1.10	R_SACCD1	true	saccharopinedehydrogenase (NADP, L-glutamateforming)	[c] : l2aadp6sa + glu_l + h + nadph <=> h2o + nadp + saccrp_l
Threonine and Lysine Metabolism	.	R_OXAGm	true	non-enzymaticreaction	[m] : h + oxag <=> 2oxoadp + co2
Threonine and Lysine Metabolism	4.2.1.79	R_MCITDm	true	_2-methyl-citratedehydratase, mitochondrial	[m] : hcit <=> b124tc + h2o
Threonine and Lysine Metabolism	6.1.1.6	R_LYSTRSm	false	Lysyl-tRNAsynthetase, mitochondrial	[m] : atp + lys_l + trnalys → amp + lystrna + ppi

Threonine and Lysine Metabolism	6.1.1.6	R_LYSTRS	false	Lysyl-tRNAsynthetase	[c] : atp + lys_l + trnalys → amp + lystrna + ppi
Threonine and Lysine Metabolism	1.1.1.155	R_HICITDm	true	homoisocitratedehydrogenase	[m] : hicit + nad <=> h + nadh + oxag
Threonine and Lysine Metabolism	4.2.1.36	R_HACNHm	true	homoacontinatehydratase, mitochondrial	[m] : b124tc + h2o <=> hicit
Threonine and Lysine Metabolism	2.6.1.39	R_AATA	true	_2-aminoadipatetransaminase	[c] : 2oxoadp + glu_l <=> l2aadp + akg
Threonine and Lysine Metabolism	1.2.1.31	R_AASAD1	false	L-aminoadipate-semialdehydedehydrogenase (NADPH)	[c] : l2aadp + atp + h + nadph → l2aadp6sa + amp + nadp + ppi
Threonine and Lysine Metabolism	1.2.1.31	R_AASAD2	false	L-aminoadipate-semialdehydedehydrogenase (NADH)	[c] : l2aadp + atp + h + nadh → l2aadp6sa + amp + nad + ppi
Threonine and Lysine Metabolism	4.2.99.2	R_4HTHRS	false	_4-Hydroxy-L-threoninesynthase	[c] : h2o + phthr → 4hthr + pi
Transport, Extracellular	.	R_VALt2r	true	L-valinereversibletransportviaprotonsymport	h[e] + val_l[e] <=> h[c] + val_l[c]
Transport, Extracellular	.	R_URIt2	false	uridinetransportinviaprotonsymport	h[e] + uri[e] → h[c] + uri[c]
Transport, Extracellular	.	R_UREA2t2	true	ureareversibletransportviaprotonsymport (2H+)	2.0 h[e] + urea[e] <=> h[c] + urea[c]
Transport, Extracellular	.	R_URAt2	false	uraciltransportinviaprotonsymport	h[e] + ura[e] → h[c] + ura[c]
Transport, Extracellular	.	R_TYRt2r	true	L-tyrosinereversibletransportviaprotonsymport	h[e] + tyr_l[e] <=> h[c] + tyr_l[c]
Transport, Extracellular	.	R_TRPt2r	true	L-tryptophanreversibletransportviaprotonsymport	h[e] + trp_l[e] <=> h[c] + trp_l[c]
Transport, Extracellular	.	R_THRt2r	true	L-threoninereversibletransportviaprotonsymport	h[e] + thr_l[e] <=> h[c] + thr_l[c]
Transport, Extracellular	.	R_THMt2	false	Thiaminetransportinviaprotonsymport	h[e] + thm[e] → h[c] + thm[c]
Transport, Extracellular	.	R_SO4ti	false	sulfateirreversibleuniport	so4[e] → so4[c]
Transport, Extracellular	.	R_SERt2r	true	L-serinereversibletransportviaprotonsymport	h[e] + ser_l[e] <=> h[c] + ser_l[c]
Transport, Extracellular	.	R_PYRt2	false	pyruvatetransportinviaprotonsymport	h[e] + pyr[e] → h[c] + pyr[c]
Transport, Extracellular	.	R_PROt2r	true	L-prolinereversibletransportviaprotonsymport	h[e] + pro_l[e] <=> h[c] + pro_l[c]
Transport, Extracellular	.	R_PNTOt2	true	Pantothenatereversibletransportviaprotonsymport	h[e] + pnto_r[e] <=> h[c] + pnto_r[c]
Transport, Extracellular	.	R_PIt2r	true	phosphatereversibletransportviasymport	h[e] + pi[e] <=> h[c] + pi[c]
Transport, Extracellular	.	R_PHEt2r	true	L-phenylalaninereversibletransportviaprotonsymport	h[e] + phe_l[e] <=> h[c] + phe_l[c]
Transport, Extracellular	.	R_ORNt2r	true	orntithinereversibletransportinviaprotonsymport	h[e] + orn[e] <=> h[c] + orn[c]
Transport, Extracellular	.	R_METt2r	true	L-methioninereversibletransportviaprotonsymport	h[e] + met_l[e] <=> h[c] + met_l[c]
Transport, Extracellular	.	R_MANt2	false	D-mannosetransportinviaprotonsymport	h[e] + man[e] → h[c] + man[c]
Transport, Extracellular	.	R_MALTt2	false	maltosetransportinviaprotonsymport	h[e] + malt[e] → h[c] + malt[c]
Transport, Extracellular	.	R_LYSt2r	true	L-lysinereversibletransportviaprotonsymport	h[e] + lys_l[e] <=> h[c] + lys_l[c]
Transport, Extracellular	.	R_LEUt2r	true	L-leucinereversibletransportviaprotonsymport	h[e] + leu_l[e] <=> h[c] + leu_l[c]
Transport, Extracellular	.	R_L_DASH_LACt2r	true	L-lactatereversibletransportviaprotonsymport	h[e] + lac_l[e] <=> h[c] + lac_l[c]
Transport, Extracellular	.	R_INSTt2	false	inositoltransportinviaprotonsymport	h[e] + inost[e] → h[c] + inost[c]
Transport, Extracellular	.	R_ILEt2r	true	L-isoleucinereversibletransportviaprotonsymport	h[e] + ile_l[e] <=> h[c] + ile_l[c]

Transport, Extracellular	.	R_HISt2r	true	L-histidinereversible-transportviaproton-symport	h[e] + his_l[e] <=> h[c] + his_l[c]
Transport, Extracellular	.	R_GUAt2r	true	guaninereversible-transportviaproton-symport	gua[e] + h[e] <=> gua[c] + h[c]
Transport, Extracellular	.	R_GTHOXti	false	oxidizedglutathioneir-reversibleuniport	gthox[e] → gthox[c]
Transport, Extracellular	.	R_GLYt2r	true	glycinereversible-transportviaproton-symport	gly[e] + h[e] <=> gly[c] + h[c]
Transport, Extracellular	.	R_GLUt2r	true	L-glutamatetransport-viaprotonsymport, reversible	glu_l[e] + h[e] <=> glu_l[c] + h[c]
Transport, Extracellular	.	R_GLNt2r	true	L-glutaminereversible-transportviaproton-symport	gln_l[e] + h[e] <=> gln_l[c] + h[c]
Transport, Extracellular	.	R_GLCt1	false	glucosetransport (uniport)	glc_d[e] → glc_d[c]
Transport, Extracellular	.	R_GALt2	false	D-galactosetransport-inviaprotonsymport	gal[e] + h[e] → gal[c] + h[c]
Transport, Extracellular	.	R_FRUt2	false	D-fructosetransport-inviaprotonsymport	fru[e] + h[e] → fru[c] + h[c]
Transport, Extracellular	.	R_CYSt2r	true	L-cysteinereversible-transportviaproton-symport	cys_l[e] + h[e] <=> cys_l[c] + h[c]
Transport, Extracellular	.	R_CSNt2	false	cytosinetransportinvia-protonsymport	csn[e] + h[e] → csn[c] + h[c]
Transport, Extracellular	.	R_CRNt	true	L-carnitinereversible-transport	crn[e] <=> crn[c]
Transport, Extracellular	.	R_BTNt2i	false	Biotinuptake	btn[e] + h[e] → btn[c] + h[c]
Transport, Extracellular	3.6.1.35	R_ATPS	false	ATPase, cytosolic	atp[c] + h2o[c] → adp[c] + h[e] + pi[c]
Transport, Extracellular	.	R_ASPt2r	true	L-aspartatereversible-transportviaproton-symport	asp_l[e] + h[e] <=> asp_l[c] + h[c]
Transport, Extracellular	.	R_ASNt2r	true	L-asparaginereversible-transportviaproton-symport	asn_l[e] + h[e] <=> asn_l[c] + h[c]
Transport, Extracellular	.	R_ARGt2r	true	L-arganinereversible-transportviaproton-symport	arg_l[e] + h[e] <=> arg_l[c] + h[c]
Transport, Extracellular	.	R_AMETt2	false	S-adenosyl-L-methioninetransport-inviaprotonsymport	amet[e] + h[e] → amet[c] + h[c]
Transport, Extracellular	.	R_ALLTTti	false	allantoateirreversible-uniport	alltt[e] → alltt[c]
Transport, Extracellular	.	R_ALLTNti	false	allantoinirreversible-uniport	alltn[e] → alltn[c]
Transport, Extracellular	.	R_ALAt2r	true	L-alaninereversible-transportviaproton-symport	ala_l[e] + h[e] <=> ala_l[c] + h[c]
Transport, Extracellular	.	R_ACt2r	true	acetatereversible-transportviaproton-symport	ac[e] + h[e] <=> ac[c] + h[c]
Transport, Extracellular	.	R_ABUTt2	false	_4-aminobutyrate-transportinviaproton-symport	4abut[e] + h[e] → 4abut[c] + h[c]
Transport, Extracellular	.	R_5AOPt2	false	_5-Aminolevulinate-transportinviaproton-symport	5aop[e] + h[e] → 5aop[c] + h[c]
Transport, Mitochondrial	.	R_VALt2m	true	Valinereversible-mitochondrialtransport-viaprotonsymport	h[c] + val_l[c] <=> h[m] + val_l[m]
Transport, Mitochondrial	.	R_TYRt2m	true	tyrosinemitochondrial-transportviaproton-symport	h[c] + tyr_l[c] <=> h[m] + tyr_l[m]
Transport, Mitochondrial	.	R_TRPt2m	true	tryptophanmitochondrial-transportviaproton-symport	h[c] + trp_l[c] <=> h[m] + trp_l[m]
Transport, Mitochondrial	.	R_THRt2m	true	threoninemitochondrial-transportviaproton-symport	h[c] + thr_l[c] <=> h[m] + thr_l[m]
Transport, Mitochondrial	.	R_SUCFUMtm	false	succinate-fumarate-transport, mitochondrial	fum[m] + succ[c] → fum[c] + succ[m]
Transport, Mitochondrial	.	R_SUCCtm	false	succinatetransport, mitochondrial	pi[m] + succ[c] → pi[c] + succ[m]
Transport, Mitochondrial	.	R_PYRt2m	true	pyruvatemitochondrial-transportviaproton-symport	h[c] + pyr[c] <=> h[m] + pyr[m]

Transport, Mitochondrial	.	R_PROtm	true	L-prolinetransport, mitochondrial	pro_l[c] <=> pro_l[m]
Transport, Mitochondrial	.	R_PIt5m	true	phosphatetransport-viahydroxideionsymport, mitochondrial	oh1[m] + pi[c] <=> oh1[c] + pi[m]
Transport, Mitochondrial	.	R_PIt2m	true	phosphatetransporter, mitochondrial	h[c] + pi[c] <=> h[m] + pi[m]
Transport, Mitochondrial	.	R_PHEt2m	true	Phenylalaninemitochondrial-transportviaproton-symport	h[m] + phe_l[m] <=> h[c] + phe_l[c]
Transport, Mitochondrial	.	R_OXO2Ctm	true	_2-oxodicarboylate-transporter, mitochondrial	akg[m] + oxag[c] <=> akg[c] + oxag[m]
Transport, Mitochondrial	.	R_ORNt3m	true	ornithinemitochondrial-transportviaproton-antiport	h[c] + orn[m] <=> h[m] + orn[c]
Transport, Mitochondrial	.	R_OAAt2m	true	oxaloacetatetransport, mitochondrial	h[c] + oaa[c] <=> h[m] + oaa[m]
Transport, Mitochondrial	.	R_METt2m	true	methioninemitochondrial-transportviaproton-symport	h[m] + met_l[m] <=> h[c] + met_l[c]
Transport, Mitochondrial	.	R_MALtm	true	malatetransport, mitochondrial	mal_l[c] + pi[m] <=> mal_l[m] + pi[c]
Transport, Mitochondrial	.	R_HISt2m	true	histidinemitochondrial-transportviaproton-symport	h[m] + his_l[m] <=> h[c] + his_l[c]
Transport, Mitochondrial	.	R_GLUt5m	false	L-glutamatetransport-intomitochondriaviahydroxideionantiport	glu_l[c] + oh1[m] → glu_l[m] + oh1[c]
Transport, Mitochondrial	.	R_GLUt2m	true	L-glutamatereversible-transportviaproton-symport, mitochondrial	glu_l[c] + h[c] <=> glu_l[m] + h[m]
Transport, Mitochondrial	.	R_FADFMNtm	false	FAD/FMNantiport	fad[c] + fmn[m] → fad[m] + fmn[c]
Transport, Mitochondrial	.	R_E4Ptm	true	D-erythrose4--phosphate-mtiochondrialtransport-viadiffusion	e4p[c] <=> e4p[m]
Transport, Mitochondrial	.	R_DICtm	true	dicarboxylatetransport, mitochondrial	mal_l[c] + succ[m] <=> mal_l[m] + succ[c]
Transport, Mitochondrial	.	R_CRNCARtm	false	carnithine-acetylcarnithinecarrier, mitochondrial	acrn[c] + crn[m] → acrn[m] + crn[c]
Transport, Mitochondrial	.	R_CITtcm	true	citratetransport, mitochondrial	cit[c] + icit[m] <=> cit[m] + icit[c]
Transport, Mitochondrial	.	R_CITtbm	true	citratetransport, mitochondrial	cit[c] + pep[m] <=> cit[m] + pep[c]
Transport, Mitochondrial	.	R_CITtam	true	citratetransport, mitochondrial	cit[c] + mal_l[m] <=> cit[m] + mal_l[c]
Transport, Mitochondrial	.	R_ATPtm_DASH_H	false	ADP/ATPtransporter, mitochondrial	adp[c] + atp[m] + h[c] → adp[m] + atp[c] + h[m]
Transport, Mitochondrial	.	R_ASPt2m	true	aspartatemitochondrial-transportviaproton-symport	asp_l[c] + h[c] <=> asp_l[m] + h[m]
Transport, Mitochondrial	.	R_ASNt2m	true	asparaginemitochondrial-transportviaproton-transport	asn_l[c] + h[c] <=> asn_l[m] + h[m]
Transport, Mitochondrial	.	R_ARGt2m	true	argininemitochondrial-transportviaproton-symport	arg_l[c] + h[c] <=> arg_l[m] + h[m]
Transport, Mitochondrial	.	R_AMETtm	true	S-Adenosyl-L-methioninereversible-transport, mitochondrial	amet[c] <=> amet[m]
Transport, Mitochondrial	.	R_AHCYStm	true	S-adenosyl-L-homocysteinereversible-transport, mitochondrial	ahcys[c] <=> ahcys[m]
Transport, Mitochondrial	.	R_ACtm	true	acetatetransport, mitochondrial	ac[c] <=> ac[m]
Transport, Mitochondrial	.	R_4ABUTNtm	true	_4-aminobutanalmitochondrial-transportviadiffusion	4abutn[c] <=> 4abutn[m]
Transport, Mitochondrial	.	R_4ABUTtm	true	_4-aminobutanoatemitochondrial-transportviadiffusion	4abut[c] <=> 4abut[m]
Transport, Mitochondrial	.	R_3MOPtm	true	_3-Methyl-2-oxopentanoate-transport, diffusion, mitochondrial	3mop[c] <=> 3mop[m]

Transport, Mitochondrial	.	R_3MOBtm	true	_3-methyl-2-oxobutanoatetransport, diffusion, mitochondrial	3mob[c] <=> 3mob[m]
Transport, Mitochondrial	.	R_3C4MOPtm	true	_3-Carboxy-4-methyl--2-oxopentanoate-transport, diffusion, mitochondrial	3c4mop[c] <=> 3c4mop[m]
Transport, Mitochondrial	.	R_3C3HMPtm	true	_2-Isopropylmalate-transport, diffusion, mitochondrial	3c3hmp[c] <=> 3c3hmp[m]
Transport, Mitochondrial	.	R_34HPPt2m	true	_3-(4-hydroxyphenyl)pyruvatemitochondrial-transportviaproton-symport	34hpp[c] + h[c] <=> 34hpp[m] + h[m]
Transport, Mitochondrial	.	R_2OXOADPtim	false	_2-oxoadipatetransport-outofmitochondriaviadiffusion	2oxoadp[m] → 2oxoadp[c]
Transport, Mitochondrial	.	R_2DDA7Ptm	true	_2-Dehydro-3-deoxy-D-arabino-heptonate7-phohsphatemitochondrial-transportviadiffusion	2dda7p[c] <=> 2dda7p[m]
Transport, Nuclear	.	R_PIt2n	true	phosphatenuclear-transportviaproton-symport	h[c] + pi[c] <=> h[n] + pi[n]
Transport, Nuclear	.	R_H2Otn	true	H2Otransport, nuclear	h2o[n] <=> h2o[c]
Transport, Nuclear	.	R_GLUt2n	true	glutamatenuclear-transportviaproton-symport	glu_l[c] + h[c] <=> glu_l[n] + h[n]
Transport, Nuclear	.	R_GLNt2n	true	glutaminenuclear-transportviaproton-symport	gln_l[c] + h[c] <=> gln_l[n] + h[n]
Transport, Nuclear	.	R_CO2tn	true	CO2nucleartransport-viadiffusion	co2[n] <=> co2[c]
Transport, Nuclear	.	R_CBPtn	true	carbamoylphosphate-nucleartransport-viadiffusion	cbp[c] <=> cbp[n]
Transport, Peroxisomal	.	R_TYRt2p	true	tyrosineperoxisomal-transportviaproton-symport	h[c] + tyr_l[c] <=> h[x] + tyr_l[x]
Transport, Peroxisomal	.	R_HCYSt2p	true	Homocysteineperoxisomal-transportviaproton-symport	h[c] + hcys_l[c] <=> h[x] + hcys_l[x]
Transport, Peroxisomal	.	R_34HPPt2p	true	_3-(4-hydroxyphenyl)pyruvateperoxisomal-transportviaproton-symport	34hpp[c] + h[c] <=> 34hpp[x] + h[x]
Transport, Peroxisomal	.	R_CYSTtp	true	cystathioneperoxisomal-transport	cyst_l[c] <=> cyst_l[x]
Tyrosine, Tryptophan, and Phenylalanine Metabolism	6.1.1.1	R_TYRTRSm	false	tyrosyl-tRNAsynthetase, mitochondrial	[m] : atp + trnatyr + tyr_l → amp + ppi + tyrtrna
Tyrosine, Tryptophan, and Phenylalanine Metabolism	6.1.1.1	R_TYRTRS	false	tyrosyl-tRNAsynthetase	[c] : atp + trnatyr + tyr_l → amp + ppi + tyrtrna
Tyrosine, Tryptophan, and Phenylalanine Metabolism	2.6.1.1	R_TYRTAp	true	tyrosinetransaminase, peroxisomal	[x] : akg + tyr_l <=> 34hpp + glu_l
Tyrosine metabolism	2.6.1.1	R_TYRTA	true	tyrosinetransaminase	[c] : akg + tyr_l <=> 34hpp + glu_l
Tyrosine metabolism	2.6.1.1	R_TYRTAm	true	tyrosinetransaminase, mitochondrial	[m] : akg + tyr_l <=> 34hpp + glu_l
Tyrosine, Tryptophan, and Phenylalanine Metabolism	2.6.1.-	R_TYRTAi	false	tyrosinetransaminase, irreversible	[c] : 34hpp + glu_l → akg + tyr_l
Tyrosine, Tryptophan, and Phenylalanine Metabolism	6.1.1.2	R_TRPTRSm	false	Tryptophanyl-tRNAsynthetase, mitochondrial	[m] : atp + trnatrp + trp_l → amp + ppi + trptrna
Tyrosine, Tryptophan, and Phenylalanine Metabolism	6.1.1.2	R_TRPTRS	false	Tryptophanyl-tRNAsynthetase	[c] : atp + trnatrp + trp_l → amp + ppi + trptrna

Tyrosine, Tryptophan, and Phenylalanine Metabolism	4.2.1.20	R_TRPS1	false	tryptophansynthase (indoleglycerol-phosphate)	[c] : 3ig3p + ser_l → g3p + h2o + trp_l
Tryptophan metabolism	1.13.11.11	R_TRPO2	false	L-Tryptophan:oxygen2, 3-oxidoreductase (decyclizing)	[c] : o2 + trp_l → lfmkynr
Tyrosine, Tryptophan, and Phenylalanine Metabolism	1.1.1.25	R_SHK3D	false	shikimatedehydrogenase	[c] : 3dhsk + h + nadph → nadp + skm
Tyrosine, Tryptophan, and Phenylalanine Metabolism	2.7.1.71	R_SHKK	false	shikimatekinase	[c] : atp + skm → adp + h + skm5p
Tyrosine, Tryptophan, and Phenylalanine Metabolism	2.5.1.19	R_PSCVTi	false	_3-phosphoshikimate1-carboxyvinyl-transferase, irreversible	[c] : pep + skm5p → 3psme + pi
Tyrosine, Tryptophan, and Phenylalanine Metabolism	5.3.1.24	R_PRAIi	false	phospho-ribosylanthranilateisomerase (irreversible)	[c] : pran → 2cpr5p
Tyrosine, Tryptophan, and Phenylalanine Metabolism	1.3.1.12	R_PPND	false	prephenatede-hydrogenase	[c] : nad + pphn → 34hpp + co2 + nadh
Tyrosine, Tryptophan, and Phenylalanine Metabolism	1.3.1.13	R_PPND2	false	prephenatede-hydrogenase (NADP)	[c] : nadp + pphn → 34hpp + co2 + nadph
Tyrosine, Tryptophan, and Phenylalanine Metabolism	4.2.1.51	R_PPNDH	false	prephenatedehydratase	[c] : h + pphn → co2 + h2o + phpyr
Tyrosine, Tryptophan, and Phenylalanine Metabolism	6.1.1.20	R_PHETRSm	false	phenylalanyl-tRNAsynthetase, mitochondrial	[m] : atp + phe_l + trnaphe → amp + phetrna + ppi
Tyrosine, Tryptophan, and Phenylalanine Metabolism	6.1.1.20	R_PHETRS	false	Phenylalanyl-tRNAsynthetase	[c] : atp + phe_l + trnaphe → amp + phetrna + ppi
Phenylalanine metabolism	2.6.1.1	R_PHETA1	true	phenylalaninetransaminase	[c] : akg + phe_l <=> glu_l + phpyr
Tryptophan metabolism	4.1.1.45	R_PCLAD	false	picolinicacidde-carboxylase	[c] : cmusa + h → am6sa + co2
Tyrosine metabolism	5.2.1.2	R_MACACI	false	maleylacetoacetateisomerase	[c] : 4mlacac → 4fumacac
Tryptophan metabolism	1.14.13.9	R_KYN3OX	false	kynurenine3-monooxygenase	[c] : lkynr + h + nadph + o2 → h2o + hlkynr + nadp
Tryptophan metabolism	3.7.1.3	R_KYN	false	kynureninase	[c] : lkynr + h2o → ala_l + anth + h
Tyrosine, Tryptophan, and Phenylalanine Metabolism	4.1.1.74	R_INDPYRD	true	Indole-3-pyruvatede-carboxylase	[c] : h + indpyr <=> co2 + id3acald
Tyrosine, Tryptophan, and Phenylalanine Metabolism	4.1.1.48	R_IGPS	false	indole-3-glycerol--phosphatesynthase	[c] : 2cpr5p + h → 3ig3p + co2 + h2o
Tryptophan metabolism	3.7.1.3	R_HKYNH	false	_3-Hydroxy-L-kynurenine-hydrolase	[c] : h2o + hlkynr → 3hanthrn + ala_l
Tyrosine metabolism	1.13.11.5	R_HGNTOR	false	Homogentisate:oxygen1, 2-oxidoreductase (decyclizing)	[c] : hgentis + o2 → 4mlacac + h
Tyrosine metabolism	3.7.1.2	R_FUMAC	false	fumarylacetoacetase	[c] : 4fumacac + h2o → acac + fum + h
Tryptophan metabolism	3.5.1.9	R_FKYNH	false	N-Formyl-L-kynurenineamido-hydrolase	[c] : lfmkynr + h2o → lkynr + for + h

Tyrosine, Tryptophan, and Phenylalanine Metabolism	4.2.1.10	R_DHQTi	false	_3-dehydroquinatedehydratase, irreversible	[c] : 3dhq → 3dhsk + h2o
Tyrosine, Tryptophan, and Phenylalanine Metabolism	4.6.1.3	R_DHQS	false	_3-dehydroquinatesynthase	[c] : 2dda7p → 3dhq + pi
Tyrosine, Tryptophan, and Phenylalanine Metabolism	4.1.2.15	R_DDPAm	false	_2-deoxy-D-arabino-heptulosonate7--phosphatesynthetase, mitochondrial	[m] : e4p + h2o + pep → 2dda7p + pi
Tyrosine, Tryptophan, and Phenylalanine Metabolism	4.1.2.15	R_DDPA	false	_3-deoxy-D-arabino-heptulosonate7--phosphatesynthetase	[c] : e4p + h2o + pep → 2dda7p + pi
Tyrosine, Tryptophan, and Phenylalanine Metabolism	4.2.3.5	R_CHORS	false	chorismatesynthase	[c] : 3psme → chor + pi
Tyrosine, Tryptophan, and Phenylalanine Metabolism	5.4.99.5	R_CHORM	false	chorismatemutase	[c] : chor → pphn
Tyrosine, Tryptophan, and Phenylalanine Metabolism	2.4.2.18	R_ANPRT	false	anthranilatephosphoribosyltransferase	[c] : anth + prpp → ppi + pran
Tyrosine, Tryptophan, and Phenylalanine Metabolism	3.5.1.4	R_AMID3	false	amidase	[c] : h2o + iad → ind3ac + nh4
Tyrosine, Tryptophan, and Phenylalanine Metabolism	3.5.1.4	R_AMID2	false	amidase	[c] : h2o + pad → nh4 + pac
Tryptophan metabolism	1.2.1.32	R_AM6SAD	false	aminomuconate-semialdehydedehydrogenase	[c] : am6sa + h2o + nad → amuco + h + nadh
Glycolysis/Gluconeogenesis	1.2.1.3	R_ALDD2y	false	aldehydedehydrogenase (acetaldehyde, NADP)	[c] : acald + h2o + nadp → ac + h + nadph
Tyrosine, Tryptophan, and Phenylalanine Metabolism	1.2.1.3	R_ALDD2ym	false	aldehydedehydrogenase (acetylaldehyde, NADP), mitochondrial	[m] : acald + h2o + nadp → ac + h + nadph
Glycolysis/Gluconeogenesis	1.2.1.3	R_ALDD2xm	false	aldehydedehydrogenase (acetylaldehyde, NAD), mitochondrial	[m] : acald + h2o + nad → ac + h + nadh
Tryptophan metabolism	1.2.1.3	R_ALDD20xm	false	aldehydedehydrogenase (indole-3-acetaldehyde, NAD), mitochondrial	[m] : h2o + id3acald + nad → h + ind3ac + nadh
Tyrosine, Tryptophan, and Phenylalanine Metabolism	1.2.1.3	R_ALDD20ym	false	aldehydedehydrogenase (indole-3-acetaldehyde, NADP), mitochondrial	[m] : h2o + id3acald + nadp → h + ind3ac + nadph
Tyrosine, Tryptophan, and Phenylalanine Metabolism	1.2.1.3	R_ALDD20y	false	aldehydedehydrogenase (indole-3-acetaldehyde, NADP)	[c] : h2o + id3acald + nadp → h + ind3ac + nadph
Phenylalanine metabolism	1.2.1.3	R_ALDD19x	false	aldehydedehydrogenase (phenylacetaldehyde, NAD)	[c] : h2o + nad + pacald → h + nadh + pac
Tryptophan metabolism	1.13.11.6	R_3HAO	false	_3-hydroxyanthranilate3, 4-dioxygenase	[c] : 3hanthrn + o2 → cmusa + h
Tyrosine metabolism	1.13.11.27	R_34HPPOR	false	_4-Hydroxyphenylpyruvate:oxygenoxidoreductase	[c] : 34hpp + o2 → co2 + hgentis

Valine, Leucine, and Isoleucine Metabolism	2.6.1.26	R_VALTA	true	valinetransaminase	[c] : akg + val_l <=> 3mob + glu_l
Valine, Leucine, and Isoleucine Metabolism	6.1.1.9	R_VALTRSm	false	valyl-tRNAsynthetase, mitochondrial	[m] : atp + trnaval + val_l → amp + ppi + valtrna
Valine, Leucine, and Isoleucine Metabolism	6.1.1.9	R_VALTRS	false	Valyl-tRNAsynthetase	[c] : atp + trnaval + val_l → amp + ppi + valtrna
Valine, Leucine, and Isoleucine Metabolism	2.6.1.42	R_OMCDCm	false	_2-oxo-4-methyl-3-carboxypentanoatedecarboxylation	[m] : 3c4mop + h → 4mop + co2
Valine, Leucine, and Isoleucine Metabolism	1.1.1.85	R_OMCDC	false	_2-Oxo-4-methyl-3-carboxypentanoatedecarboxylation	[c] : 3c4mop + h → 4mop + co2
Valine, Leucine, and Isoleucine Metabolism	6.1.1.4	R_LEUTRS	false	Leucyl-tRNAsynthetase	[c] : atp + leu_l + trnaleu → amp + leutrna + ppi
Valine, Leucine, and Isoleucine Metabolism	6.1.1.4	R_LEUTRSm	false	leucyl-tRNAsynthetase, mitochondrial	[m] : atp + leu_l + trnaleu → amp + leutrna + ppi
Valine, Leucine, and Isoleucine Metabolism	2.6.1.26	R_LEUTA	true	leucinetransaminase	[c] : akg + leu_l <=> 4mop + glu_l
Valine, Leucine, and Isoleucine Metabolism	2.6.1.26	R_LEUTAm	true	leucinetransaminase, mitochondrial	[m] : akg + leu_l <=> 4mop + glu_l
Valine, Leucine, and Isoleucine Metabolism	1.1.1.86	R_KARA1im	false	acetohydroxyacidisomero-reductase, mitochondrial	[m] : alac_s + h + nadph → 23dhmb + nadp
Valine, Leucine, and Isoleucine Metabolism	1.1.1.86	R_KARA2im	false	ketol-acidreductoisomerase (2-Aceto-2-hydroxybutanoate), mitochondrial	[m] : 2ahbut + h + nadph → 23dhmp + nadp
Valine, Leucine, and Isoleucine Metabolism	4.1.3.12	R_IPPSm	false	_2-isopropylmalatesynthase, mitochondrial	[m] : 3mob + accoa + h2o → 3c3hmp + coa + h
Valine, Leucine, and Isoleucine Metabolism	4.1.3.12	R_IPPS	false	_2-isopropylmalatesynthase	[c] : 3mob + accoa + h2o → 3c3hmp + coa + h
Valine, Leucine, and Isoleucine Metabolism	4.2.1.33	R_IPPMIb	true	_2-isopropylmalatehydratase	[c] : 2ippm + h2o <=> 3c3hmp
Valine, Leucine, and Isoleucine Metabolism	4.2.1.33	R_IPPMIa	true	_3-isopropylmalatedehydratase	[c] : 3c2hmp <=> 2ippm + h2o
Valine, Leucine, and Isoleucine Metabolism	1.1.1.85	R_IPMD	false	_3-isopropylmalatedehydrogenase	[c] : 3c2hmp + nad → 3c4mop + h + nadh
Valine, Leucine, and Isoleucine Metabolism	2.6.1.26	R_ILETA	true	isoleucinetransaminase	[c] : akg + ile_l <=> 3mop + glu_l
Valine, Leucine, and Isoleucine Metabolism	2.6.1.26	R_ILETAm	true	isoleucinetransaminase, mitochondrial	[m] : akg + ile_l <=> 3mop + glu_l
Valine, Leucine, and Isoleucine Metabolism	6.1.1.5	R_ILETRSm	false	isoleucyl-tRNAsynthetase, mitochondrial	[m] : atp + ile_l + trnaile → amp + iletrna + ppi

Valine, Leucine, and Isoleucine Metabolism	6.1.1.5	R_ILETRS	false	Isoleucyl-tRNAsynthetase	[c] : atp + ile_l + trnaile → amp + iletrna + ppi
Valine, Leucine, and Isoleucine Metabolism	4.2.1.9	R_DHAD2m	false	dihydroxy-aciddehydratase (2, 3-dihydroxy-3-methyl-pentanoate), mitochondrial	[m] : 23dhmp → 3mop + h2o
Valine, Leucine, and Isoleucine Metabolism	4.2.1.9	R_DHAD1m	false	dihydroxy-aciddehydratase (2, 3-dihydroxy-3-methyl-butanoate), mitochondrial	[m] : 23dhmb → 3mob + h2o
Valine, Leucine, and Isoleucine Metabolism	4.1.3.18	R_ACLSm	false	acetolactatesynthase, mitochondrial	[m] : h + 2.0 pyr → alac_s + co2
Valine, Leucine, and Isoleucine Metabolism	4.1.3.18	R_ACHBSm	false	_2-aceto-2-hydroxybutanoatesynthase, mitochondrial	[m] : 2obut + h + pyr → 2ahbut + co2
Xylose Metabolism	1.-.-.-	R_XYLR	false	xylosereductase	[c] : h + nadph + xyl_d → nadp + xylt
Xylose Metabolism	1.1.1.9	R_XYLTD_D	false	xylitoldehydrogenase (D-xyulose-forming)	[c] : nad + xylt → h + nadh + xylu_d
Nucleotides	2.7.1.20	R_ADNK1	false	adenosinekinase	[c] : adn + atp → adp + amp + h
NAD Metabolism	2.4.2.19	R_NNDPR	false	nicotinate-nucleotide-diphosphorylase (carboxylating)	[c] : 2 h + prpp + quln → co2 + nicrnt + ppi
NAD Biosynthesis	2.4.2.11	R_NAPRTr	true	NAPRTase (rev)	[c] : h + nac + prpp ⇔ nicrnt + ppi
NAD Biosynthesis	2.4.2.11	R_NAPRTm	false	NAPRTase, mitochondrial	[m] : h + nac + prpp → nicrnt + ppi
Cofactor and Prosthetic Group Biosynthesis	2.4.2.11	R_NAMNPP	false	nicotinicacidmononucleotidepyrophosphorylase	[c] : atp + h2o + nac + prpp → adp + nicrnt + pi + ppi
Salvage Pathway	2.4.2.7	R_ADPT	false	adeninephosphoribosyltransferase	[c] : ade + prpp → amp + ppi
T	3.5.1.5	R_UREA	false	urease	[c] : 2 h + h2o + urea → co2 + 2 nh4
Putative Transporters	.	R_ALLTNt2r	true	allantointransportinviaprotonsymport	alltn[e] + h[e] ⇔ alltn[c] + h[c]
Transport, Extracellular	.	R_ADEt	true	adeninereversible-transport, cytosol	ade[e] ⇔ ade[c]
Transport, Peroxisomal	.	R_NMNt2p	true	NMNperoxisomal-transportviaproton-symport	h[c] + nmn[c] ⇔ h[x] + nmn[x]
Transport, Peroxisomal	.	R_H2Otp	true	NMNperoxisomal-transportviaproton-symport	h2o[c] ⇔ h2o[x]
Transport, Extracellular	.	R_NCAMUP	false	Nicotinamideaciduptake	ncam[e] → ncam[c]

Table S1: Low-Cost Exchange Reactions

Low-Cost Sources	ala-L^e, arg-L^e, asn-L^e, asp-L^e, cys-L^e, gln-L^e, glu-L^e, gly^e, his-L^e, ile-L^e, leu-L^e, lys-L^e, met-L^e, phe-L^e, pro-L^e, ser-L^e, thr-L^e, trp-L^e, tyr-L^e, val-L^e, ade^e, adn^e, cytd^e, csn^e, gua^e, gsn^e, thym^e, thymd^e, ura^e, uri^e, hxan^e, xan^e, ins^e, a-D-glucose^e, b-D-glucose^e, glc-D^e, rib-D^e, drib^e, h2o^e, co2^e, o2^e, nh4^e, hco3^e, pi^e, fe2^e, fe3^e, so4^e, ni2^e, k^e, na1^e, mn2^e, cl^e, cu2^e, ca2^e, zn2^e, mg2^e, cd2^e, mobd^e, s^e, tcynt^e, urate^e, orot^e, ptrc^e, sprm^e, spmd^e, amet^e, gthrd^e, inost^e, chol^e, orn^e, pheme^e, avite1^e, btn^e, ascb-L^e, thm^e, pydx^e, pydxn^e, vitd2^e, 4abz^e, ribflv^e, retinol^e, thf^e, fol^e, fmn^e, nmn^e, ncam^e, nac^e, nad^e, coa^e, pnto-R^e, chsterol^e, ergst^e, zymst^e, ac^e, succ^e, lac-D^e, fum^e, mal-L^e, lac-L^e, pyr^e, fru^e, strch1^e, cellobiose^e, gal^e, sucr^e, xyl-D^e, lcts^e, xylt^e, man^e, arab-D^e, malt^e, strch2^e, cellulose^e, malttr^e
Low-Cost Escapes	h2o^e, co2^e, nh4^e, hco3^e, orot^e, ac^e, succ^e, lac-D^e, h^e, fum^e, mal-L^e, lac-L^e, acac^e, for^e, acetone^e, urea^e, acald^e, pyr^e, etoh^e

Table S2: Results of First Round of FBA-Gap

Organism	$\lvert R \rvert$	$\lvert M \rvert$	Minimum Biomass	Sources Selected	Escapes Selected	Biomass Flux	Notes
bsu	1251	1224	0.5	P_i^e, Ca_2^e, Mg_2^e, Cys-L^e, Fe_3^e, $Malttr^e$, K^e, O_2^e, NH_4^e	Lac-L^e	0.5	cost: 10.0
T. cruzi	112	130	0.5	b-D-$glucose^e$, Glu-L^e, Asp-L^e, P_i^e	$Succinate^e$, H^e, NH_4^e	0.5	cost: 7.0
hpy	552	563	0.5	Ile-L^e, Val-L^e, Cys-L^e, O_2^e, Ala-L^e, Met-L^e, His-L^e, Leu-L^e, P_i^e, Gly^e, Orn^e	CO_2^e, Lac-L^e, NH_4^e, $rhcys^c$	0.5	cost: 18214.0
ecoli	1075	908	0.5	$Cytd^e$, O_2^e, Cys-L^e	Clpn_EC^c, Lac-d^e, Ura^e	18.0	cost: 12925.0
crypto	576	712	0.5	AMP^c, $NADP^c$, Asp-L^e, $AcCoA^c$, $s17bp^c$, SO_4^e, Ser-L^e, Pyr^e, Gln-L^e	CO_2^c, Ala-L^e	6.2	cost: 526.0

Table S3: Metabolites that are distance ∞ from biomass reaction for *C. neoformans*

NotSources	13bdglcn^c, 2dr5p^c, 2omhmbl_5^m, 2ommbl_5^m, 3ophb_5^c, 3ophb_5^m, 4aabutn^c, 4adcho^c, 4gudbutn^c, 4h2oglt^c, 4h2oglt^m, 4h2oglt^x, 4hpro_lt^c, 4hthr^c, 5aop^m, 5aprbu^c, 5fthf^m, 5mta^c, abt^c, acac^c, acoa^x, acrn^x, ade^c, adp^v, ahdt^c, alatrna^c, amp^x, amuco^c, apep^c, argtrna^c, argtrna^m, asntrna^c, asntrna^m, asptrna^c, asptrna^m, atp^v, cgly^c, chitos^c, cholp^c, cit^x, clpn_sc^m, cmaphis^c, co2^g, co2^v, co2^x, coa^n, crn^c, crn^e, cystrna^c, damp^c, dara14lac^c, dhf^c, dhnpt^c, dolmanp^r, dolp^r, dtmp^c, duri^c, ethamp^c, fgam^c, fmettrna^m, fmn^c, gam6p^c, gdp^g, gdpmann^c, gdpmann^g, glntrna^c, gluala^c, glutrna^c, glutrna^m, glytrna^c, gthrd^m, h2o^v, h^g, h^r, h^v, hcit^n, histrna^c, histrna^m, hmbil^c, hpglu^c, iletrna^c, iletrna^m, ind3ac^c, ind3ac^m, inost^c, lac_d^c, leutrna^c, leutrna^m, lystrna^c, lystrna^m, m2macchitppdol^g, m3macchitppdol^g, m4macchitppdol^g, mannan^r, mettrna^c, mi145p^c, mi1p_d^c, nadph^x, nh4^e, nh4^x, nmn^x, npmehis^c, pac^c, pant-r^m, paps^c, pc_sc^c, pe_sc^g, pe_sc^m, pe_sc^v, phetrna^c, phetrna^m, pi^v, ppi^x, pppg9^c, pram^c, protrna^c, ps_sc^g, ps_sc^v, ptd3ino_sc^c, ptd4ino_sc^c, ptd4ino_sc^n, pyr^x, ribflv^e, sertrna^c, spmd^c, srb_l^c, tglp^c, thm^c, thmmp^c, thrtrna^c, thrtrna^m, trdrd^c, trptrna^c, trptrna^m, tyrtrna^c, tyrtrna^m, valtrna^c, valtrna^m, xylu_d^c
NotEscapes	10fthf^m, 13bdglcn^e, 1ag3p_sc^c, 1p3h5c^c, 25dhpp^c, 2dhp^m, 2omhmbl_5^m, 2ommbl_5^m, 35ccmp^c, 35cdamp^c, 35cgmp^c, 35cimp^c, 3ophb_5^m, 4abut^e, 4abutn^c, 4abutn^m, 4ahmmp^c, 4gudbd^c, 4hbz^m, 4hpro_lt^m, 4mhetz^c, 5aop^e, 5mthf^c, 6dg^c, ac^x, acald^m, accoa^n, acrn^c, acybut^c, adp^v, akg^n, ala-b^c, alltn^e, alltt^e, amet^e, aprop^c, arab-d^c, arab-l^c, asp-l^n, atp^x, btn^e, caphis^c, cdpchol^c, ch4s^c, chol^c, co2^g, co2^x, crn^x, csn^e, cytd^c, dagpy_sc^c, dcacoa^c, dcyt^c, ddcacoa^c, dhpmp^c, dolmanp^r, dolp^r, drib^c, e4hglu^c, e4hglu^x, etha^c, fad^c, fmn^c, fmn^e, fmn^m, fru^e, gal^e, gar^c, gdp^g, gdpmann^g, glc-d^e, glp^c, glx^x, gsn^c, gthox^e, h2o^e, h2o^x, h2s^c, h^g, h^r, hdcoa^c, hxan^c, iad^c, icit^x, id3acald^m, ind3acnl^c, inost^e, lgt_s^m, lys-l^m, m2macchitppdol^g, m3macchitppdol^g, m4macchitppdol^g, malt^e, man^e, mannan^r, methf^m, mhpglu^c, mthgxl^c, n4abutn^c, nadp^x, nadph^x, nicrnt^c, nicrnt^m, nmn^c, nmn^m, o2^m, ocdycacoa^c, octdp_5^c, octdp_5^m, odecoa^c, pacald^c, pad^c, pant-r^c, pap^c, pe_sc^g, pepd^c, pg_sc^m, phthr^c, pi^v, pmtcoa^c, ps_sc^g, ps_sc^v, ptd145bp_sc^c, ptd1ino_sc^c, ptd1ino_sc^n, pyr^e, ribflv^e, sbt-l^c, so4^c, so4^e, stcoa^c, sucr^e, tdcoa^c, tglp^c, thm^e, thmmp^e, thmpp^e, trdox^c, trnaala^c, trnaarg^c, trnaarg^m, trnaasn^c, trnaasn^m, trnaasp^c, trnaasp^m, trnacys^c, trnagln^c, trnaglu^c, trnaglu^m, trnagly^c, trnahis^c, trnahis^m, trnaile^c, trnaile^m, trnaleu^c, trnaleu^m, trnalys^c, trnalys^m, trnamet^c, trnamet^m, trnaphe^c, trnaphe^m, trnapro^c, trnaser^c, trnathr^c, trnathr^m, trnatrp^c, trnatrp^m, trnatyr^c, trnatyr^m, trnaval^c, trnaval^m, uppg3^c, ura^e, uri^e, xan^c, xyl-d^c

Table S4: Round 1 of Gap Analysis for *C. neoformans*

Metabolite	Source/Escape	Reactions Added
coa^c	source	None
$arg\text{-}l^e$	source	None
$gln\text{-}l^e$	source	None
gly^e	source	None
pyr^e	source	None
$nicrnt^c$	source	R_NNDPR, R_NAPRTr, R_NAPRTm, R_NAMNPP
$camp^c$	source	added R_ADNK1
$asp\text{-}l^e$	source	None
$so4^e$	source	None
ade^c	escape	R_ADPT, R_ADEt
$alltn^c$	escape	R_ALLNt2r
h^e	escape	None
$pro\text{-}l^e$	escape	None
$urea^e$	escape	R_UREA
$ala\text{-}l^e$	escape	None

Table S5: Round 2 of Gap Analysis for *C. neoformans*

Metabolite	Source/Escape	Reactions added
$amet^e$	source	None
coa^c	source	None
pi^e	source	None
$asn\text{-}l^e$	source	None
$gln\text{-}l^e$	source	None
gly^e	source	None
$urea^e$	source	None
pyr^e	source	None
nmn^c	source	R_NMNt2p, R_H2Otp
$so4^e$	source	None
$met\text{-}l^e$	escape	None
ade^e	escape	None
$allttn^e$	escape	None
$ala\text{-}l^e$	escape	None
$asp\text{-}l^e$	escape	None

Table S6: Round 3 of Gap Analysis for *C. neoformans*

Metabolite	Source/Escape	Reactions added
$amet^e$	source	None
coa^c	source	None
pi^e	source	None
$asn\text{-}l^e$	source	None
$gln\text{-}l^e$	source	None
gly^e	source	None
$urea^e$	source	None
pyr^e	source	None
$so4^e$	source	None
$dnad^c$	source	R_NCAMUP
$met\text{-}l^e$	escape	None
ade^e	escape	None
$allttn^e$	escape	None
$ala\text{-}l^e$	escape	None
$asp\text{-}l^e$	escape	None

Table S7: Round 4 of Gap Analysis for *C. neoformans*

Metabolite	Source/Escape	Reactions added
$amet^e$	source	None
coa^c	source	None
pi^e	source	None
$arg\text{-}l^e$	source	None
$gln\text{-}l^e$	source	None
gly^e	source	None
pyr^e	source	None
$ncam^e$	source	None
$asp\text{-}l^e$	source	None
$so4^e$	source	None
$met\text{-}l^e$	escape	None
ade^e	escape	None
$allttn^e$	escape	None
h^e	escape	None
$pro\text{-}l^e$	escape	None
$urea^e$	escape	None
$ala\text{-}l^e$	escape	None
